# BRAF^V600E^ Mutation-Responsive miRNA-222-3p Promotes Metastasis of Papillary Thyroid Cancer Cells *via* Snail-Induced EMT

**DOI:** 10.3389/fendo.2022.843334

**Published:** 2022-05-16

**Authors:** Yuan Gao, Dapeng Xiang, Weijie Li, Xi Zheng, Lin Wang, Zhiyu Li, Ting Chen

**Affiliations:** ^1^ Cancer Institute, Key Laboratory of Cancer Prevention and Intervention, Ministry of Education, Second Affiliated Hospital of Zhejiang University School of Medicine, Hangzhou, China; ^2^ Department of General Surgery, Second Affiliated Hospital School of Medicine, Zhejiang University, Hangzhou, China; ^3^ Key Laboratory of Respiratory Disease of Zhejiang Province, Department of Respiratory and Critical Care Medicine, Second Affiliated Hospital of Zhejiang University School of Medicine, Hangzhou, China; ^4^ Zhejiang Provincial Key Laboratory of Pancreatic Disease, The First Affiliated Hospital, Zhejiang University School of Medicine, Hangzhou, China

**Keywords:** papillary thyroid carcinoma, lymph node metastasis, miR-222-3p, BRAF^V600E^, Snail

## Abstract

*BRAF* mutation accounts for 50% of the PTC (papillary thyroid carcinoma) and is closely associated with high-risk clinicopathological characteristics. Increasing evidence implied that dysregulation of miRNA participated in carcinogenesis and progression of cancer. Clinical data showed the significant up-regulation of miR-222-3p in PTC; however, the role of miR-222-3p and possible relationship with *BRAF* mutation remained unclear. Here, we identified significant up-regulation of miR-222-3p in PTC tissues harboring *BRAF^V600E^* mutation compared with BRAF wild type (*BRAF^WT^*) from collected PTC clinical samples. External validation performed with The Cancer Genome Atlas (TCGA) databases was consistent with the above result. Exogenous expression of BRAF^V600E^ oncoprotein increased the expression of miR-222-3p in B-CPAP and TPC-1 cells. The treatment of BRAF^V600E^ and MEK inhibitor, PLX4720 and PD0325901, decreased the expression of miR-222-3p in B-CPAP but not in TPC-1. Inhibition of miR-222-3p significantly suppressed the migration of B-CPAP and induced a mesenchymal-epithelial transition (MET) phenotype *via* the Snail transcription factor. Immunohistochemistry (IHC) analysis demonstrated the up-regulation of Snail correlated with lymph node metastasis and *BRAF^V600E^* mutation in PTC. Besides, *in situ* hybridization (ISH) and IHC analysis of PTC clinical samples confirmed the correlation between the expression of miR-222-3p and Snail. These results showed miR-222-3p conduced more aggressive clinical manifestation of PTC by promoting Snail-induced EMT.

## Introduction

The incidence of PTC has significantly increased in the last decade worldwide. Although most PTCs have a good prognosis, some patients still went through local recurrence or distant metastasis after surgery and radioactive iodine therapy. Treatment of advanced PTC patients characterized by recurrent disease, distant metastasis, and resistance to radioactive iodine, including traditional radiotherapy, chemotherapy, and kinase inhibitors, has limited benefits, but a lot of side effects. These patients showed a poor quality of life and a dismal lifespan (5-year disease-specific survival is ~70%) (1, 2).

miRNA is one endogenous non-coding RNA with a length of ~21 nucleotides, which can promote or inhibit protein expression by combining with target mRNA following the principle of base complementary pairing (3). Aberrant miRNA expression can activate multiple oncogenic pathways in different types of cancers (4). Previous studies showed that miR-222-3p was one of the most consistently overexpressed miRNAs in PTC and an eight to eleven folds increase compared to normal thyroid tissues has been reported (5, 6). Up-regulated miR-222-3p can promote prostate cancer cell cycle transformation, enhance chordoma cell migration and facilitate endometrial carcinoma cell invasion (7–9). In PTC, the up-regulation of miR-222-3p is correlated with high-risk features such as extrathyroidal extension, lymph node metastasis, and recurrence (5, 10, 11). However, the mechanism of the metastasis and invasion conducted by dysregulation of miR-222-3p still needs to be further explored.

There are multiple driver mutations in PTC, including but not limited to *BRAF* mutation, *RAS* mutation, *TP53* mutation, *RET/PTC* rearrangement, and *NTRK1* rearrangement (12), among which the *BRAF^V600E^* mutation accounts for the highest proportion, reaching ~50%. BRAF^V600E^ oncoprotein, caused by a c.1799 T>A transversion, is associated with aggressive clinicopathologic features, such as extrathyroidal invasion, lymph node metastasis, and advanced disease stages (13, 14). Induction of *BRAF^V600E^* mutation and simultaneous activation of thyroid-stimulating hormone receptor signaling pathway can transform normal thyroid follicular cells into cancer cells (15). The BRAF^V600E^ oncoprotein could constitutively activate the mitogen-activated protein kinase (MAPK) pathway, thereby enhancing malignancy (16).

Here, by analyzing the data of PTC patients from TCGA, we found that the miR-222-3p expression was related to the *BRAF^V600E^* mutation in PTC. We supposed that BRAF^V600E^ could augment the metastasis capability of PTC by upregulating miR-222-3p expression. In this study, we verified the correlation between the *BRAF^V600E^* mutation and the up-regulation of miR-222-3p in the tumor samples of PTC patients. By adjusting the expression of BRAF^V600E^ protein in the PTC cell line, the corresponding expression level of miR-222-3p was determined. Furthermore, we tried to elaborate on the metastasis-promoting effect of miR-222-3p through the Snail-induced EMT. The clarification of the correlation between miR-222-3p and BRAF^V600E^ and the metastasis-promoting role of miR-222-3p in PTC may shed more light on the diagnosis and treatment of PTC.

## Materials and Methods

### Human Thyroid Tissues

70 cases of PTCs were collected by the Department of Pathology of Second Affiliated Hospital of Zhejiang University School of Medicine from 2019 to 2020. All PTC samples were surgically resected and the whole hematoxylin and eosin slides of all samples were reviewed by two senior pathologists. Formalin-fixed paraffin-embedded (FFPE) samples of PTCs were stored at room temperature (51 cases with *BRAF^V600E^* mutation and 19 cases with BRAF^WT^. 45 cases with lymph node metastasis and 25 cases with no metastasis).45 consecutive cases with lymph node metastasis were included in two years to compare the miR-222-3p in PTC tumors with and without lymph node metastasis.

This study was approved by the Human Research Ethics Committee of the Second Affiliated Hospital of Zhejiang University School of Medicine, Hangzhou, China. The IRB protocol number is 2019010.

### Cell Culture

B-CPAP (RRID: CVCL_0153) cell line was purchased from the Chinese Academy of Sciences (Shanghai, China). TPC-1 (RRID: CVCL_6298) cell line was purchased from Procell (Wuhan, China). K1 (RRID: CVCL_2537) cell line was purchased from Cellcook (Guangzhou, China). B-CPAP and TPC-1 were cultured in RPMI-1640 medium (Sigma), K1 was cultured in DMEM medium (Gibco). Both media were supplemented with 10% (v/v) fetal bovine serum. All cells were maintained at 5% CO_2_ and 37°C in a humidified atmosphere.

### miRNA Inhibitor Transfection and MAPK Pathway Inhibitor

Cells were seeded in a 12-well plate. After 24h, transfection was performed with Lipofectamine 3000 reagent (Thermo Fisher Scientific), following the manufacturer’s instructions. Transfection experiments were performed using 100nM miR-222-3p inhibitors or negative control (Genepharma). The inhibitor efficiency was greater than ninety-five percent. Sequences of miR-222-3p inhibitor and negative control were as following:

hsa-miR-222-3p inhibitor: 5’-ACCCAGUAGCCAGAUGUAGCU-3’; has-miR-222-3p negative control: 5’-CAGUACUUUUGUGUAGUACAA-3’.

PLX4720 (BRAF^V600E^ inhibitor) and PD0325901 (MEK inhibitor) were purchased from Selleck.

### Construction of BRAF^V600E^ Overexpression Plasmid

pcDNA3.1- plasmid was used as the vector plasmid, and Xba I (Takara, *TCTAGA*) and Hind III (Takara, *AAGCTT*) were determined as restriction sites. A *BRAF^V600E^* point mutation (GTG→GAG) and the amplification primer sequence were shown below:

BRAF forward: 5’-GC*TCTAGA*ATGGCGGCGCTGAGCGGTGG-3’, BRAF^V600E^ reverse: 5’-CTCCATCGAGATTTCTCTGTAGCTAGACCAA-3’; BRAF^V600E^ forward: 5’-TTGGTCTAGCTACAGAGAAATCTCGATGGAG-3’, BRAF reverse: 5’-CCC*AAGCTT*TCACTTGTCATCGTCGTCCTTGTAATCG TGGACAGGAAACGCACCATATC-3’.

### RNA Extraction and Quantitative Real-Time PCR

Total RNA was isolated with RNA-Quick Purification Kit (YiShan Biotech) according to the manufacturer’s instructions. Reverse transcription of mRNA and miRNA (stem-loop primer) was performed following the manufacturer’s protocols of the PrimeScript™ RT reagent kit (Takara). Quantitative real-time PCR was performed following the manufacturer’s protocols of TB Green™ *Premix Ex Taq*™ II (Takara). Expression of miR-222-3p was normalized to internal control U6. Primer sequences as following: miR-222-3p forward: 5’-GGGAGCTACATCTGGCTA-3’ and reverse: 5’-GTGTCGTGGAGTCGGCAA-3’; U6 forward: 5’-CTCGCTTCGGCAGCACA-3’ and reverse: 5’-AACGCTTCACGAATTTGCGT-3’; miR-222-3p stem-loop primer: 5’- CTCAACTGGTGTCGTGGAGTCGGCAATTCAGTTGAGGAGACC-3’.

### Western Blotting

Cells were lysed with Mammalian Protein Extract Reagent (Thermo Scientific) supplemented with protease inhibitor cocktail (1:100) (Thermo Scientific). Cell lysates were electrophoresed on 10% SDS-PAGE and then transferred to PVDF membranes. The membranes were blocked with 5% milk and then hybridized with a primary antibody at 4°C overnight. The membrane was washed and hybridized with horseradish peroxidase (HRP)-conjugated secondary antibody. Signals were visualized by SuperSignal™ West Pico PLUS Chemiluminescent Substrate (Thermo Scientific). Primary antibodies were listed below: anti-E-cadherin (HUABIO, EM0502, 1:1000), anti-Snail (HUABIO, ER1706-22, 1:4000), anti-Zeb2 (HUABIO, ER64964, 1:1000), anti-NF-κB-P65 (HUABIO, ET1603-12, 1:2000), anti-p-P65(S536) (CST, EP2294Y, 1:1000), anti-p-P65(S468) (ABclonal, AP0446, 1:1000), anti-β-actin (HUABIO, M1210-2, 1:5000), anti-DYKDDDDK Tag (HUABIO, M1402-2, 1:2000).

### Cell Migration

For transwell migration assay, cell migration ability was measured by transwell permeable chamber with 8.0 μm pore size (Corning Costar, USA). Cell transfected with miR-222-3p inhibitor or negative control were collected after 72 h. 5 × 10^4^ cells were seeded on the upper chamber. 2.5% FBS was used as a chemokine in the lower chambers. After 4-5 h of incubation at cell incubator cells were fixed with 4% paraformaldehyde for 30 min and stained with crystal violet solution for 5 min. Cells were removed from the upper membrane with cotton swabs and migrated cells on the lower membrane were taken photos under a microscope (Random 5 fields, 40 ×).

### MiRNA ISH

In brief, FFPE tissue sections were deparaffinized in xylene and rehydrated in a series of decreasing alcohol concentrations. To expose miR-222-3p probes (MiR-222-3p probe sequence: 5’-ACCCAGTAGCCAGATGTAGC-3’), tissue sections were incubated with 15 μg/mL Proteinase K solution at 37°C for 10 min, after a titration experiment that established the optimal unmasking treatment while maintaining tissue morphology. 3% methanol-H_2_O_2_ were used to block endogenous peroxidase. Tissues were then pre-hybridized with ISH buffer for 30 min, and then hybridized with locked nucleic acid-based digoxigenin (DIG)-labeled miR binding oligonucleotides at 50°C. Tissues were then washed stringently in decreasing concentrations of saline-sodium citrate (SSC) buffer. Following the stringent wash, tissues were blocked in 2% sheep serum and 1% bovine serum albumin (BSA). Tissues were then incubated with anti-DIG-HRP at 37°C for 40 min. Freshly prepared DAB color developing solution was added and the color development time was controlled under the microscope. The sections were re-dyed by Harris hematoxylin for about 3 minutes and dehydrated. Positive is brownish yellow, and the nucleus is blue.

The scoring system (1-12) is done according to the depth of the color and the proportion of the positive cell. The images corresponding to scores 0, 3, 6, 9, and 12 are shown in **Supplementary Figure 1**.

### IHC

FFPE tissue sections were deparaffinized, rehydrated, antigen retrieved using Proteinase K, and blocked in sheep serum and BSA solution as described above in the ISH section. Snail antigen was retrieved by citrate antigen retrieval solution (Beyotime Biotechnology). Then, tissue sections were incubated with polyclonal rabbit anti-human primary antibody (Snail 1:150, ER1706-22, HUABIO) at RT for 1 h. A DAB Horseradish Peroxidase Color Development Kit (Beyotime Biotechnology) was used for the subsequent steps. Tissue endogenous peroxidase activity was blocked with peroxidase block for 5 min. Thereafter, sections were incubated with horseradish peroxidase (HRP)-conjugated secondary antibody for 30 min and visualized with diaminobenzidine (DAB) substrate at RT.

### Statistical Analysis

A two-tailed *t*-test was used for continuous variables (GraphPad Prism v9.0 and SPSS v25). Mann-Whitney *t*-test was used for ranked variables. Ranked correlation analysis was used to analyze the correlation of ISH score (hsa-miR-222-3p) and IHC score (Snail) in 60 PTC samples. Each sample was corresponding to an ISH score and an H-score. The bioinformatics data on the miRNA expression and mutation status of PTC were publicly available from UCSC Xena Browser under GDC TCGA Thyroid Cancer (THCA) datasets. PTC samples in TCGA were divided into two groups (BRAF^WT^ and BRAF^V600E^ mutation) according to BRAF gene status. A two-tailed *t-*test was used for comparing the miRNA expression. *p*<0.05 was considered as statistically significant.

## Results

### The Expression of miR-222-3p in PTCs With or Without *BRAF^V600E^* Mutation

**Figure 1:**
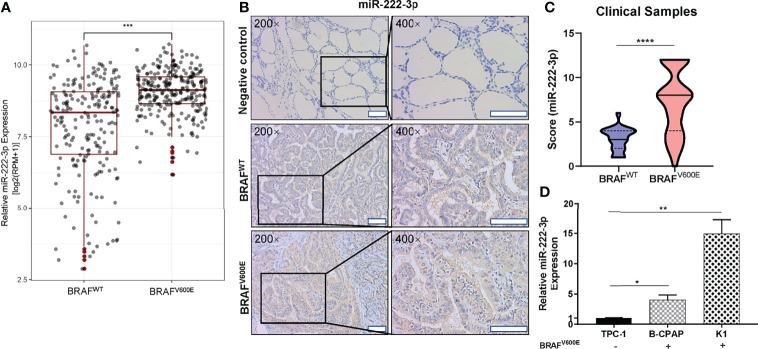
Hsa-miR-222-3p is overexpressed in PTC cells and patients harboring *BRAF^V600E^* mutation. **(A)** Differential analysis of miR-222-3p expression for 491 thyroid cancerous tissues with *BRAF^WT^* and *BRAF^V600E^* mutation. RPM: reads per million mapped reads. ***p < 0.001. **(B)** Representative images (200×, 400×) of CISH staining of PTC lesions from clinical patients harboring *BRAF^WT^* and *BRAF^V600E^* mutation. Negative control from normal thyroid tissues. Scale bar, 100 μM. **(C)** Mann-Whitney *t*-test analysis of miR-222-3p ISH score in PTC lesions between *BRAF^WT^* and *BRAF^V600E^*. The solid line and dotted line respectively represent median and interquartile range, ****p < 0.0001. **(D)** q-PCR validation of miR-222-3p expression in PTC cell lines including TPC-1, B-CPAP, and K1. Bars represent means ± SD of 3 independent experiments each measured in triplicate. *p < 0.05; **p < 0.01.

Through differential analysis of miR-222-3p expression in PTC clinical samples from TCGA, we found miR-222-3p had a higher expression level in PTC patients harboring *BRAF^V600E^* mutation (n=279) compared with that in PTC patients harboring *BRAF^WT^* (n=212) (p<0.001) (**Figure 1A**). Subsequently, to further verify the clinical correlation of miR-222-3p, we evaluated miR-222-3p expression in PTC clinical samples from our research center through ISH analysis (**Table 1**). ISH analysis of PTC patients harboring *BRAF^V600E^* mutation (n=51) and *BRAF^WT^* (n=19) demonstrated that PTC lesions with *BRAF^V600E^* mutation showed a higher level of miR-222-3p than that with *BRAF^WT^* (p<0.0001) (**Figures 1B, C**). In addition, we tested the expression of miR-222-3p in PTC cell lines, named TPC-1, B-CPAP, and K1 (TPC-1 cell line harbors *BRAF^WT^*, while B-CPAP and K1 cell line harbor *BRAF^V600E^* mutation). Through relative quantitative PCR (qPCR), we found the expression of miR-222-3p in B-CPAP (p=0.0154) and K1 (p=0.0035) cell lines was higher than that in TPC-1 (**Figure 1D**). We found that miR-222-3p was also overexpressed in the PTC cell line harboring *BRAF^V600E^* mutation.

**Table 1:** Association between miR-222-3p expression and clinicopathological features in PTC.

Clinicopathological features		n	miR-222-3p score, median (interquartile range)	*P* value
Age	<55	54	5 (3-8)	*p=0.3162*
	≥55	16	7 (4-8)	
Sex	Male	28	7 (4-8)	*p=0.5126*
	Female	42	4 (3-8.25)	
Multicentricity	No	44	7 (3-9)	*p=0.3176*
	Yes	24	5 (3-8)	
Extrathyroidal extension	No	62	6 (3-8)	*p=0.9306*
	Yes	6	5 (4-9)	
	Unknown	2	/	/
Mutation status	BRAF^WT^	19	3 (2-4)	*p < 0.0001*
	BRAF^V600E^	51	8 (4-12)	
TNM stage	I	57	6 (3-8)	*p=0.2626*
	II/III/IV	13	8 (4-10)	
T category	Tx	2	/	/
	T1/T2	48	5 (2.25-8)	*p=0.1017*
	T3/T4	20	6 (4-12)	
N category	N0	25	3 (2-4)	*p < 0.0001*
	N1	45	8 (4-12)	
M category	M0	69	6 (3-8)	/
	M1	1	4	

TNM, tumor node metastasis;. TNM, stage according to the eighth edition of AJCC.

### BRAF^V600E^ Oncoprotein Promotes miR-222-3p Expression in PTCs

**Figure 2:**
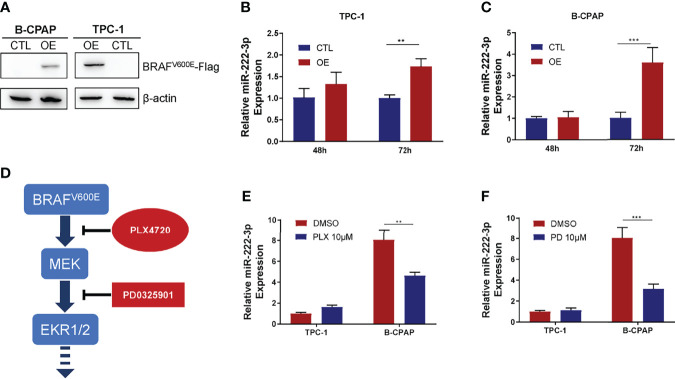
BRAF^V600E^ oncoprotein regulates miR-222-3p expression in PTCs. **(A)** immunoblot of BRAF^V600E^-Flag protein from extracts of TPC-1 and B-CPAP transfected with OE plasmid or empty vector. **(B, C)** q-PCR validation of expression level of miR-222-3p in TPC-1 and B-CPAP transfected with OE plasmid or empty vector after 48 h and 72 h. Bars represent means ± SD of 3 independent experiments each measured in triplicate. **p < 0.01; ***p < 0.001. **(D)** Model of inhibitors suppresses BRAF^V600E^-MEK-ERK pathway. **(E)** q-PCR validation of expression level of miR-222-3p in B-CPAP and TPC-1 treated with 10 μM PLX4720 or DMSO after 24 h. Bars represent means ± SD of 3 independent experiments each measured in triplicate. **p < 0.01. **(F)** q-PCR validation of expression level of miR-222-3p in B-CPAP and TPC-1 treated with 10 μM PD0325901 or DMSO after 24 h. Bars represent means ± SD of 3 independent experiments each measured in triplicate. ***p < 0.001.

The above experiments showed that miR-222-3p expression in PTC was correlated with *BRAF^V600E^* mutation. We speculated that the BRAF^V600E^ oncoprotein might promote miR-222-3p expression in PTC. To verify this hypothesis, we constructed a BRAF^V600E^-Flag overexpression plasmid (OE) with pcDNA3.1- as a vector. 48 h after transfection, the differential expression of miR-222-3p could not be observed in TPC-1 transfected with OE plasmid (OE group) and empty vector (CTL group), neither was in B-CPAP (**Figures 2A–C**). However, 72 h after transfection, miR-222-3p expression in the OE group in both TPC-1 (p=0.0069) and B-CPAP (p=0.0385) were significantly increased compared with that in the CTL group (**Figures 2B, C**). These results confirmed that the BRAF^V600E^ oncoprotein up-regulated the expression of miR-222-3p in PTC cell lines.

To further clarify how the BRAF^V600E^ oncoprotein regulates miR-222-3p, RAF/ERK pathway inhibitors were used to treat PTC cell lines and the expression of miR-222-3p was measured. PLX4720 is a highly selective BRAF^V600E^ inhibitor, and its affinity to BRAF^V600E^ mutant protein is about ten times higher than that to BRAF wild-type protein. PD0325901 is a non-ATP-competitive MEK inhibitor that inhibits the phosphorylation of ERK1 and ERK2 (**Figure 2D**). We treated TPC-1, K1, and B-CPAP with 10μM PLX4720 and PD0325901, respectively, and measured the expression of miR-222-3p by qPCR after 24 h of treatment. These inhibitors did not affect the expression of miR-222-3p in TPC-1 (**Figures 2E, F**). For the K1 cell line, we found that 1μM PD0325901 significantly reduced the expression of miR-222-3p by 70% (p=0.0308). However, 1~10μM PLX4720 didn’t inhibit the level of miR-222-3p in K1 (**Supplementary Figures 2A, B**). After treatment of B-CPAP with PLX4720, the expression of miR-222-3p was reduced by about 50% (p=0.0433), and after treatment with PD0325901, the expression of miR-222-3p in B-CPAP was reduced by about 70% (p=0.0234) (**Figures 2E, F**). These results showed that the constitutive expression of miR-222-3p in TPC-1, K1, and B-CPAP could be distinct. The RAF/MEK/ERK pathway is responsible for the expression of miR-222-3p in B-CPAP; however, it seems that BRAF^V600E^ inhibitor PLX4720 plays no effect on the activation of the MAPK/ERK pathway and miR-222-3p expression in K1. As for TPC-1, there could be other mechanisms regulating miR-222-3p expression instead of MAPK/ERK pathway.

### MiR-222-3p Is Associated With Lymph Node Metastasis in PTCs

**Figure 3:**
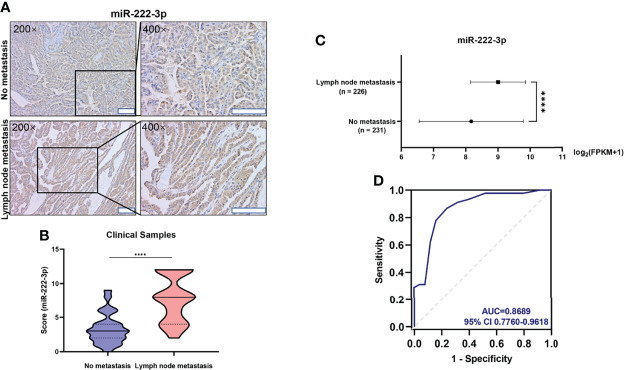
miR-222-3p is correlated with lymph node metastasis in PTCs **(A)** Representative images (200×, 400×) of CISH staining of PTC lesions from clinical patients with and without lymph node metastasis. Scale bar, 100 μM. **(B)** Mann-Whitney *t*-test analysis of miR-222-3p ISH score in PTC lesions between “No metastasis” and “lymph node metastasis” group. The solid line and dotted line respectively represent median and interquartile range, ****p < 0.0001. **(C)** Differential analysis of miR-222-3p expression between PTCs with lymph node metastasis (n = 226) and without metastasis (n = 231). FPKM: Fragments Per Kilobase per Million, ****p < 0.0001. **(D)** ROC curve for the prediction of lymph node metastasis in PTCs.

Through the above experimental data, the relationship between miR-222-3p expression and BRAF^V600E^ has been clarified. The ISH analysis also showed that PTCs with lymph node metastasis (n=45) had a higher level of miR-222-3p expression (p<0.0001) than those without lymph node metastasis (n=25) (**Figures 3A, B**). Besides, analysis of the TCGA database implied that the expression of miR-222-3p in PTCs with lymph node metastasis (n=226) was higher than that in PTCs without metastasis (n=231) (p < 0.0001) (**Figure 3C**). These results indicated that miR-222-3p overexpression might increase the risk of lymph node metastasis. Furthermore, miR-222-3p ISH score and BRAF mutation status were used to predict lymph node metastasis of PTCs. In the ROC curve analysis (**Figure 3D**), the area under the curve (AUC) was 0.8689, and the optimal cut-off value (0.5859) exhibited a sensitivity and specificity of 0.8667 and 0.76, respectively.

### Downregulation of miR-222-3p Suppresses Cell Migration and EMT of PTCs

**Figure 4:**
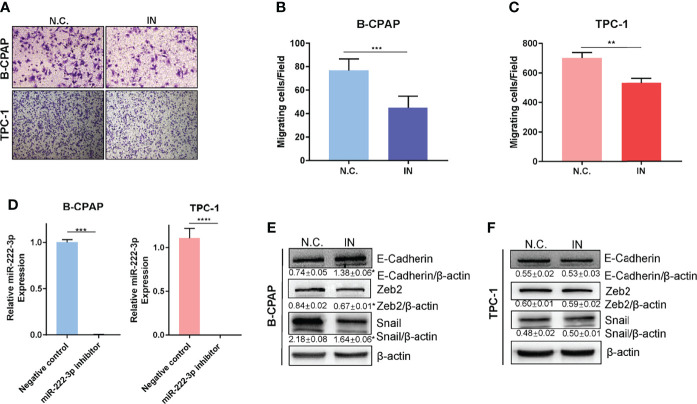
Downregulation of miR-222-3p suppresses cell migration and EMT of PTCs. **(A)** Migration images of representative microscope fields (40x, 100x). **(B, C)** *t*-test analysis of the number of B-CPAP and TPC-1 migrating cells of N.C. and IN group. *** p< 0.001,**p < 0.01. **(D)** q-PCR validation of expression level of miR-222-3p in B-CPAP and TPC-1 transfected with negative control (N.C.) and miR-222-3p inhibitor (IN). ***p < 0.001,****p < 0.0001. **(E, F)** immunoblot of E-cadherin, Zeb2, and Snail protein from extracts of B-CPAP and TPC-1 transfected N.C. and IN. *p < 0.05.

Next, we examined the role of miR-222-3p in PTC metastasis. The miR-222-3p inhibitor efficiency is greater than ninety-five percent (**Figure 4D** and **Supplementary Figure 2C**). Suppression of miR-222-3p in B-CPAP and TPC-1 cells by transfection of inhibitors suppressed cell migration (p=0.0009, p=0.0037) (**Figures 4A–C**). Nonetheless, the cell migration ability of K1 cell was not influenced by the miR-222-3p inhibitor (**Supplementary Figure 2D**). Meanwhile, EMT-related markers were measured including E-cadherin, N-cadherin, Twist1, Vimentin, Zeb1, Zeb2, Snail, and Slug through immunoblotting analysis. The decreased expression of Snail and Zeb2 and increased expression of E-cadherin were observed in the B-CPAP inhibitor group (**Figure 4E** and **Supplementary Figure 3A**). However, these changes cannot be observed in the TPC-1 cell line (**Figure 4F** and **Supplementary Figure 3B**). These results implicated that miR-222-3p influenced the cell migration ability and the underlying mechanism varied from types of cell lines or gene mutation.

### Expression of Snail Is Correlated With Lymph Node Metastasis in PTCs Harboring *BRAF^V600E^* Mutation and miR-222-3p

**Figure 5:**
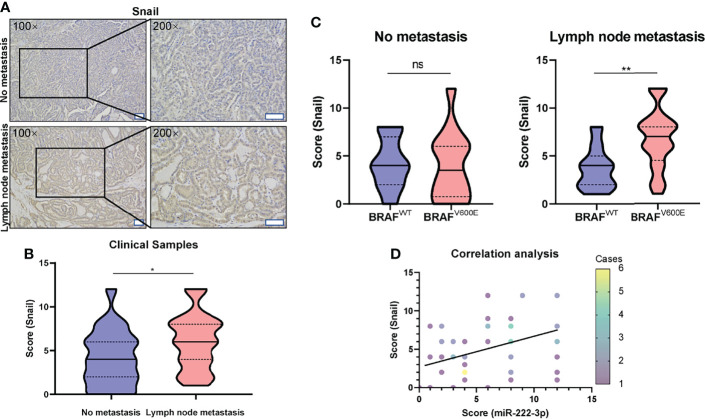
High expression of Snail is correlated with lymph node metastasis of PTC harboring *BRAF^V600E^* mutation. **(A)** Representative images (100×, 200×) of IHC staining of PTC lesions from clinical patients with and without lymph node metastasis. Scale bar, 100 μM. **(B)** Mann-Whitney *t*-test analysis of Snail H-score in PTC lesions between “No metastasis” and “lymph node metastasis” group. The solid line and dotted line respectively represent median and interquartile range, *p < 0.05. **(C)** Mann-Whitney *t*-test analysis of Snail H-score in PTC lesions with lymph node metastasis (right panel) or without metastasis (left panel) between BRAF^WT^ and BRAF^V600E^ group. The solid line and dotted line respectively represent the median and interquartile range, ns, p > 0.05, **p < 0.01. **(D)** Rank correlation analysis between the score of miR-222-3p and Snail from PTC clinical patients (Spearman r=0.33, N = 60, p=0.0111). Different color data points represented the different case numbers.

*In vitro* experiment, we showed that miR-222-3p regulated the expression of Snail in B-CPAP. We further examined the expression of Snail in 60 clinical PTC samples by IHC. Through rank correlation analysis, we found that, Snail expression was correlated with miR-222-3p in the PTC clinical samples (r=0.33, p=0.0111) (**Figure 5D**).

Furthermore, we also analyzed the correlation between Snail expression and PTC lymph node metastasis. The Snail IHC score in the tumor tissue of PTC patients with lymph node metastasis was significantly higher than that without lymph node metastasis (p=0.0219) (**Figures 5A, B**). Furthermore, in the tumor tissues of PTC patients without lymph node metastasis, *BRAF^V600E^* mutation is not significantly related to the Snail IHC score (**Figure 5C** left); while in the tumor tissues of PTC patients with lymph node metastasis, Snail IHC score in the *BRAF^V600E^* mutation group was significantly higher than that in the BRAF^WT^ group (p=0.0072) (**Figure 5C** right). Consistent with the rank correlation analysis above, PTCs with both lymph node metastasis and *BRAF^V600E^* mutation had the highest Snail IHC score and the highest miR-222-3p ISH score among the four groups. Taken together, these results showed that miR-222-3p could promote lymph node metastasis through upregulating Snail in PTCs harboring *BRAF^V600E^* mutation.

## Discussion

In this study, we clarified the correlation between the *BRAF^V600E^* mutation and the overexpression of miR-222-3p in PTC. Meanwhile, we demonstrated that the miR-222-3p could promote the EMT *via* up-regulation of Snail and lymph node metastasis of PTCs.

Previous studies related to this subject were either focused on the correlation analysis between the *BRAF^V600E^* mutation and aggressive clinicopathological characteristics such as recurrence, local extrathyroidal invasion, lymph node metastasis, and distant metastasis (17) or concentrated on the dysregulation of miR-222-3p in PTC patients (10). However, there is a lack of exploration between the *BRAF^V600E^* mutation and the high expression of miR-222-3p in PTCs. In the present study, we used the public TCGA database for big data analysis combined with small sample verification to clarify that the *BRAF^V600E^* mutation in PTC is related to the upregulation of miR-222-3p expression. Here, the expression level of miR-222-3p in clinical PTC samples was measured *via* the semi-quantitative CISH because of the small tumor size and the inevitable false positive staining of red blood cells in FISH. By inhibiting BRAF^V600E^ and MEK, or overexpressing exogenous BRAF^V600E^ in PTC cell lines, we confirmed that the expression level of miR-222-3p was indeed regulated by the *BRAF^V600E^* mutation and BRAF-MEK-ERK pathway. In another word, miR-222-3p is located downstream of the BRAF^V600E^/ERK pathway and may act as an intermediate molecule of BRAF^V600E^ oncoprotein to promote the malignant transformation. A similar conclusion was drawn in breast cancer that miR-222-3p was downstream of RAS/RAF/MEK/ERK (18).

Aberrant expression of miR-222-3p played an essential role in various types of cancers. Fan et al. found that decreased miR-222-3p promoted EMT in epithelial ovarian cancer (19). However, up-regulation of miR-222-3p in cancers was likely to be aggressive characteristics including promotion of cell proliferation, inhibition of cell apoptosis, and enhancement of cell migration and invasion (18, 20). Gulluoglu et al. found that increased miR-222-3p promoted chordoma progression by targeting E-cadherin-mediated Zeb1-induced EMT (9). In breast cancer, up-regulation of miR-222-3p promoted Zeb2-induced EMT by targeting transcriptional repressor TRPS1 (18). Here, we found that inhibition of miR-222-3p could suppress EMT-related transcription factors, Snail and Zeb2, and meanwhile suppress cell migration in PTCs. MiR-222-3p could also facilitate or complicate migration and invasion in several types of cancers such as osteosarcoma, gastric cancer, and endometrial carcinoma (7, 21, 22). ISH analysis and IHC analysis of PTC clinical samples also revealed the correlation between miR-222-3p and Snail. Previous studies demonstrated the regulation of BRAF^V600E^ on Snail in thyroid cancer cell lines and the correlation between *SNAI1* mRNA expression and lymph node metastasis in PTC patients (23, 24). Here, using IHC analysis of PTC clinical samples, we demonstrated that the expression of Snail in cancerous tissues was related to lymph node metastasis from the protein level, especially in patients harboring *BRAF^V600E^* mutation. In summary, our study demonstrated that the regulation of miR-222-3p by BRAF^V600E^ oncoprotein promoted cell migration of PTCs as well as Snail-induced EMT and was correlated with lymph node metastasis of PTC patients.

Aberrant expression of microRNAs in the patients’ body fluids could be a piece of novel evidence to assist diagnosis of thyroid carcinoma and post-operation monitoring. On the basic findings of this topic, more studies needed to be carried out to validate our results. Meanwhile, the molecular mechanisms under how miR-222-3p affect lymph node metastasis of PTC patients also need to be further analyzed. These promising leads will provide a new potential biomarker for both diagnosis and prognosis in the clinic.

## Data Availability Statement

The original contributions presented in the study are included in the article/**Supplementary Material**. Further inquiries can be directed to the corresponding author.

## Ethics Statement 

The studies involving human participants were reviewed and approved by Human Research Ethics Committee of the Second Affiliated Hospital of Zhejiang University School of Medicine, Hangzhou, China. The patients/participants provided their written informed consent to participate in this study.

## Author Contributions

Conception and design: TC, YG, and DX. Collection and assembly of the data: DX, ZL, LW, and YG. Language editing and grammar correction: TC. Development of the methodology: WL and XZ. Data analysis and interpretation: all authors. Manuscript writing: all authors. All authors contributed to the article and approved the submitted version.

## Funding

The present study was funded by the National Natural Science Foundation of China (grant no. 81500115) and the Science and Technology Department of Zhejiang Province (grant no. 2016C33141).

## Conflict of Interest

The authors declare that the research was conducted in the absence of any commercial or financial relationships that could be construed as a potential conflict of interest.

## Publisher’s Note

All claims expressed in this article are solely those of the authors and do not necessarily represent those of their affiliated organizations, or those of the publisher, the editors and the reviewers. Any product that may be evaluated in this article, or claim that may be made by its manufacturer, is not guaranteed or endorsed by the publisher.

## Supplementary Material

Supplementary Figure 1 The images of the miR-222-3p ISH assay corresponding to scores 0, 3, 6, 9, and 12. Scale bar, 100 μM

Supplementary Figure 2 The regulation of miR-222-3p in K1 cell line and cell migration experiment. **(A)** q-PCR validation of expression level of miR-222-3p in K1 treated with 1~10 μM PD0325901 or DMSO after 24 h. Bars represent means ± SD of 3 independent experiments each measured in triplicate. *p < 0.05. **(B)** q-PCR validation of expression level of miR-222-3p in K1 treated with 1~10 μM PLX4720 or DMSO after 24 h. Bars represent means ± SD of 3 independent experiments each measured in triplicate. **(C)** q-PCR validation of expression level of miR-222-3p in K1 transfected with N.C and IN. **p < 0.01. **(D)** Migration images of random microscope fields (200x). There is no significant cell number difference between N.C. and IN in K1.

Supplementary Figure 3 Statistical analysis graphs of EMT-associated proteins expression between N.C. and IN. **(A)** Using student’s t-test method to compare the expression level of E-cadherin, Zeb2, and Snail between N.C. and IN in B-CPAP cell line. *p < 0.05. **(B)** Using student’s t-test method to compare the expression level of E-cadherin, Zeb2, and Snail between N.C. and IN in TPC-1 cell line.
