# Supplementary figures and images for: BRAFV600E Mutation-Responsive miRNA-222-3p Promotes Metastasis of Papillary Thyroid Cancer Cells via Snail-Induced EMT
Source: Front Endocrinol (Lausanne). 2022 May 16;13:843334. doi: 10.3389/fendo.2022.843334 (PMC9148970; doi:10.3389/fendo.2022.843334)

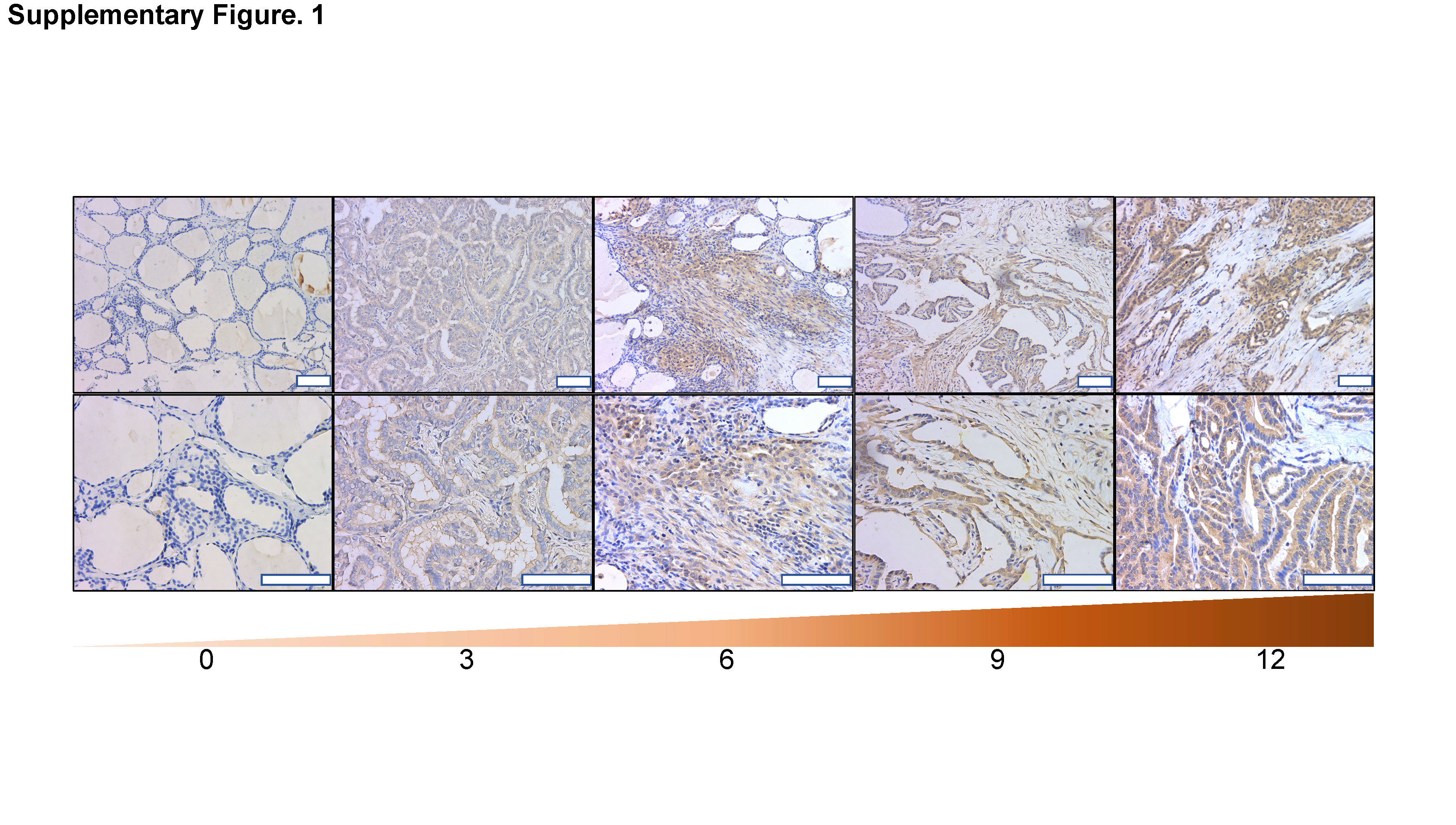

Supplement: Supplementary Figure 1 — The images of the miR-222-3p ISH assay corresponding to scores 0, 3, 6, 9, and 12. Scale bar, 100 μM [file Image_1.jpeg]

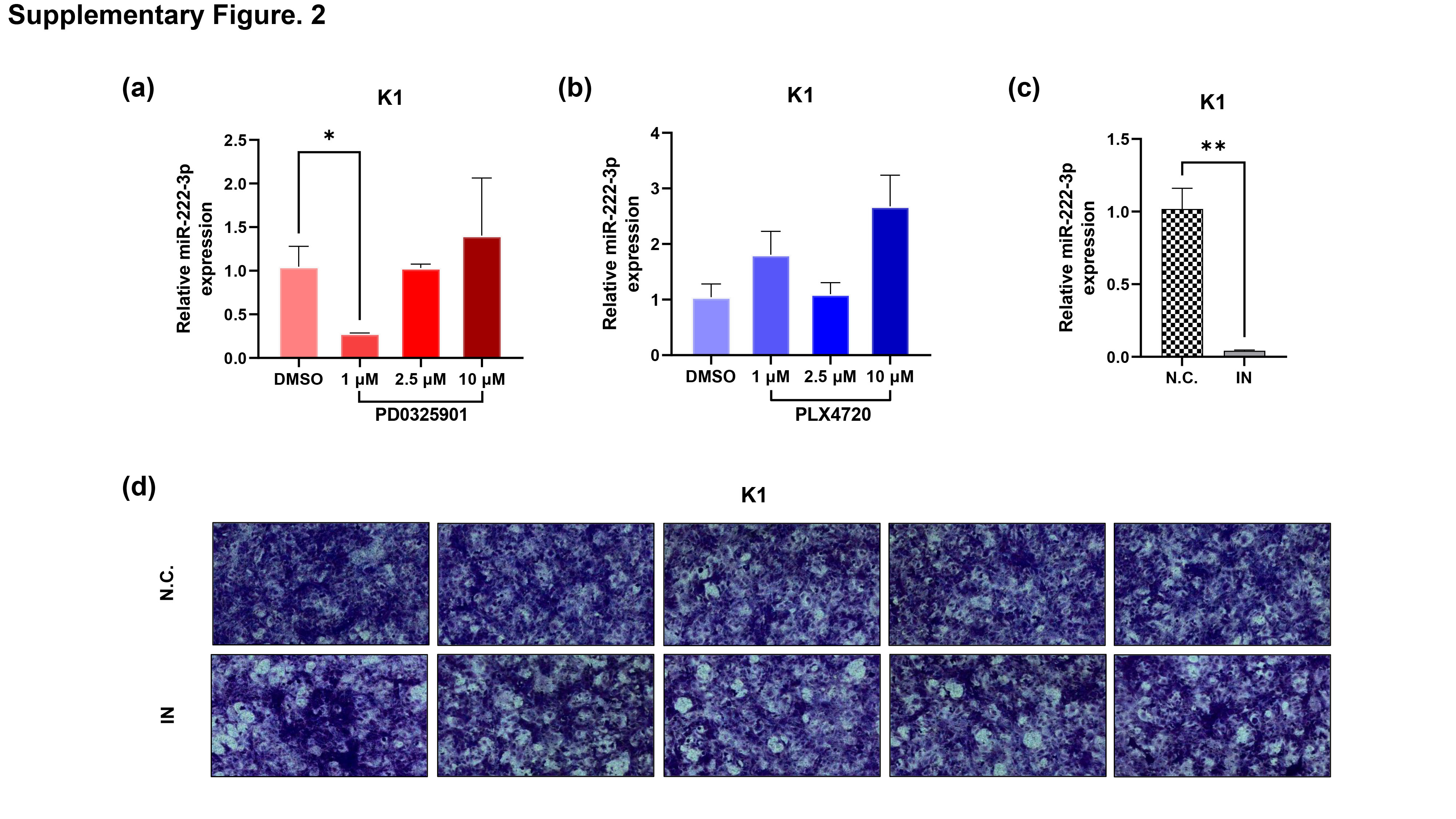

Supplement: Supplementary Figure 2 — The regulation of miR-222-3p in K1 cell line and cell migration experiment. (A) q-PCR validation of expression level of miR-222-3p in K1 treated with 1~10 μM PD0325901 or DMSO after 24 h. Bars represent means ± SD of 3 independent experiments each measured in triplicate. *p < 0.05. (B) q-PCR validation of expression level of miR-222-3p in K1 treated with 1~10 μM PLX4720 or DMSO after 24 h. Bars represent means ± SD of 3 independent experiments each measured in triplicate. (C) q-PCR validation of expression level of miR-222-3p in K1 transfected with N.C and IN. **p < 0.01. (D) Migration images of random microscope fields (200x). There is no significant cell number difference between N.C. and IN in K1. [file Image_2.jpeg]

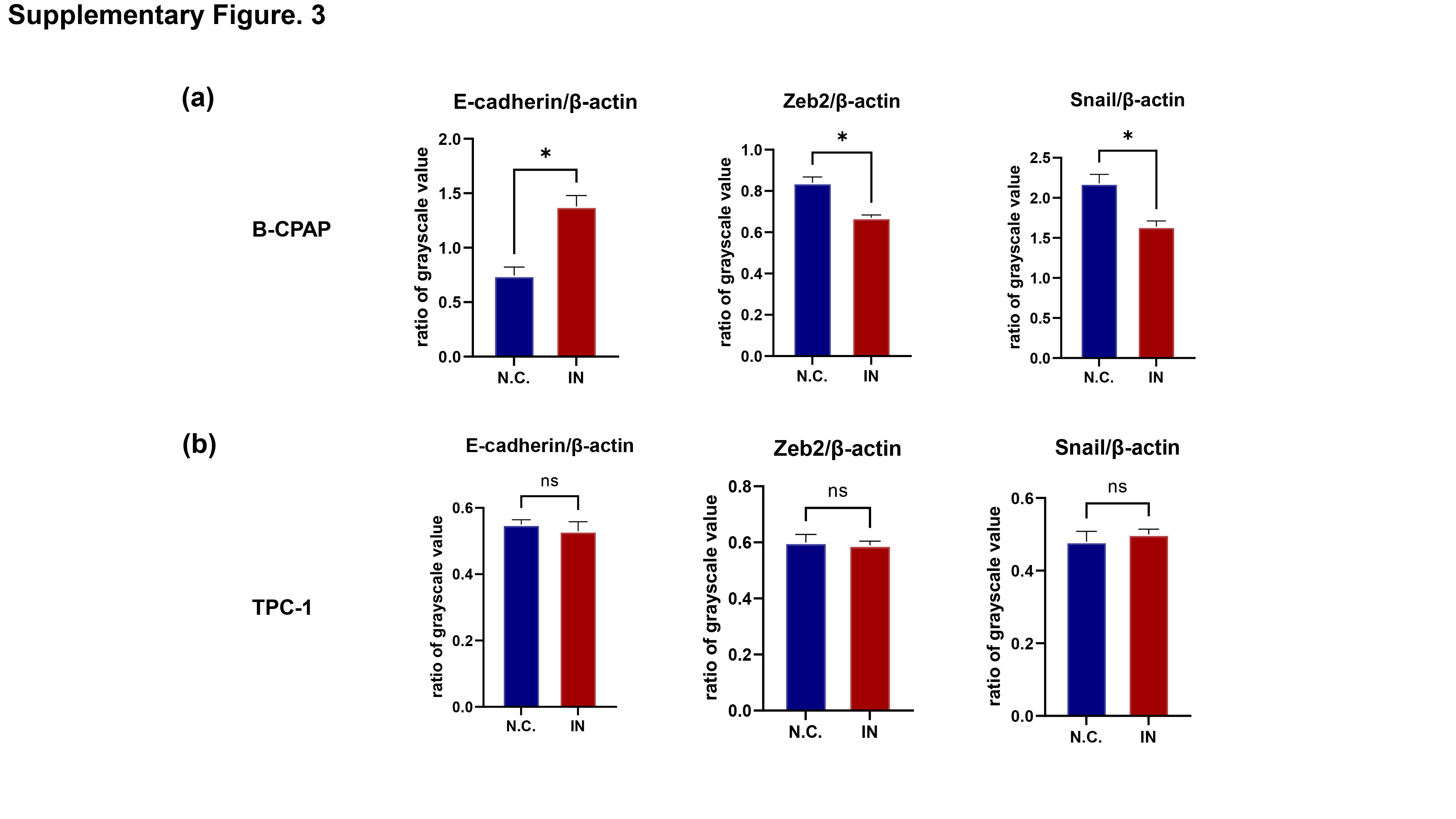

Supplement: Supplementary Figure 3 — Statistical analysis graphs of EMT-associated proteins expression between N.C. and IN. (A) Using student’s t-test method to compare the expression level of E-cadherin, Zeb2, and Snail between N.C. and IN in B-CPAP cell line. *p < 0.05. (B) Using student’s t-test method to compare the expression level of E-cadherin, Zeb2, and Snail between N.C. and IN in TPC-1 cell line. [file Image_3.jpeg]

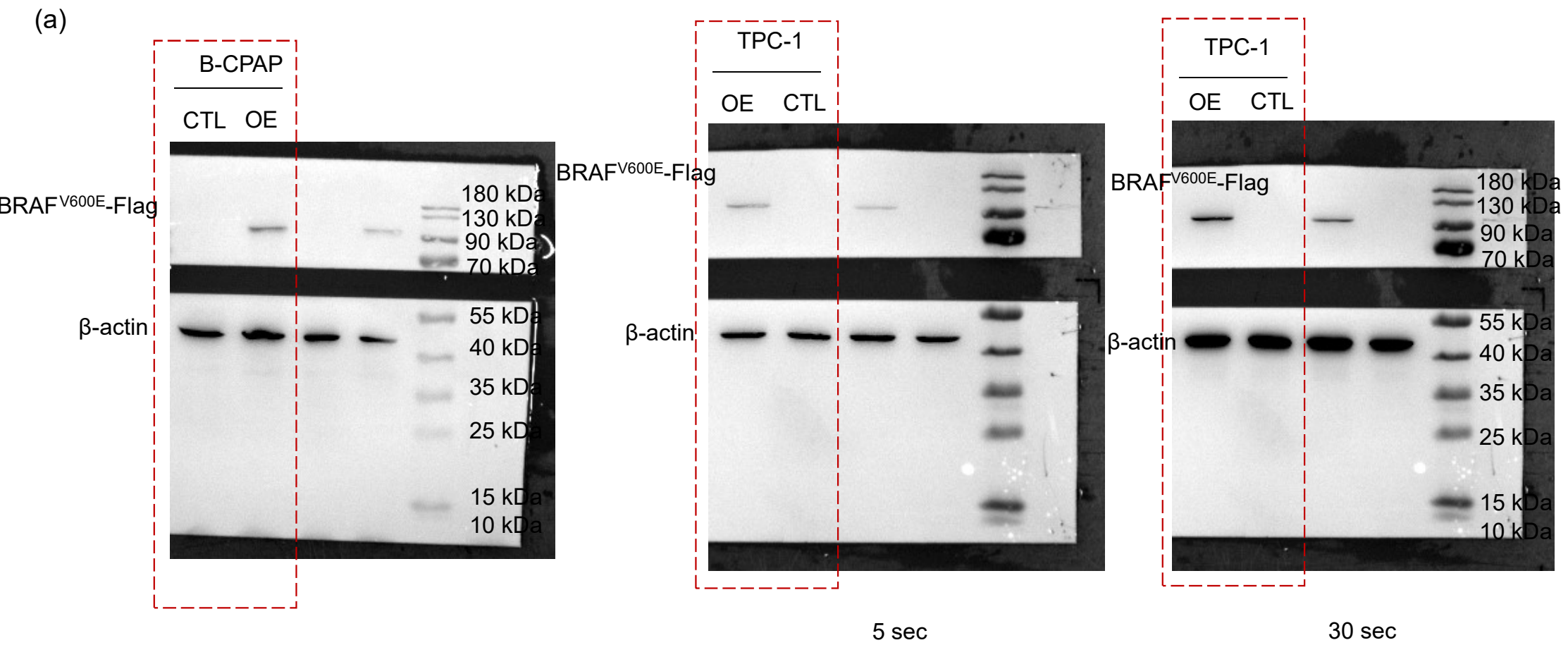

Fig. 4 original blot images

PageRuler™ Prestained Protein Ladder, 10 to 180 kDa, Cat: 26616

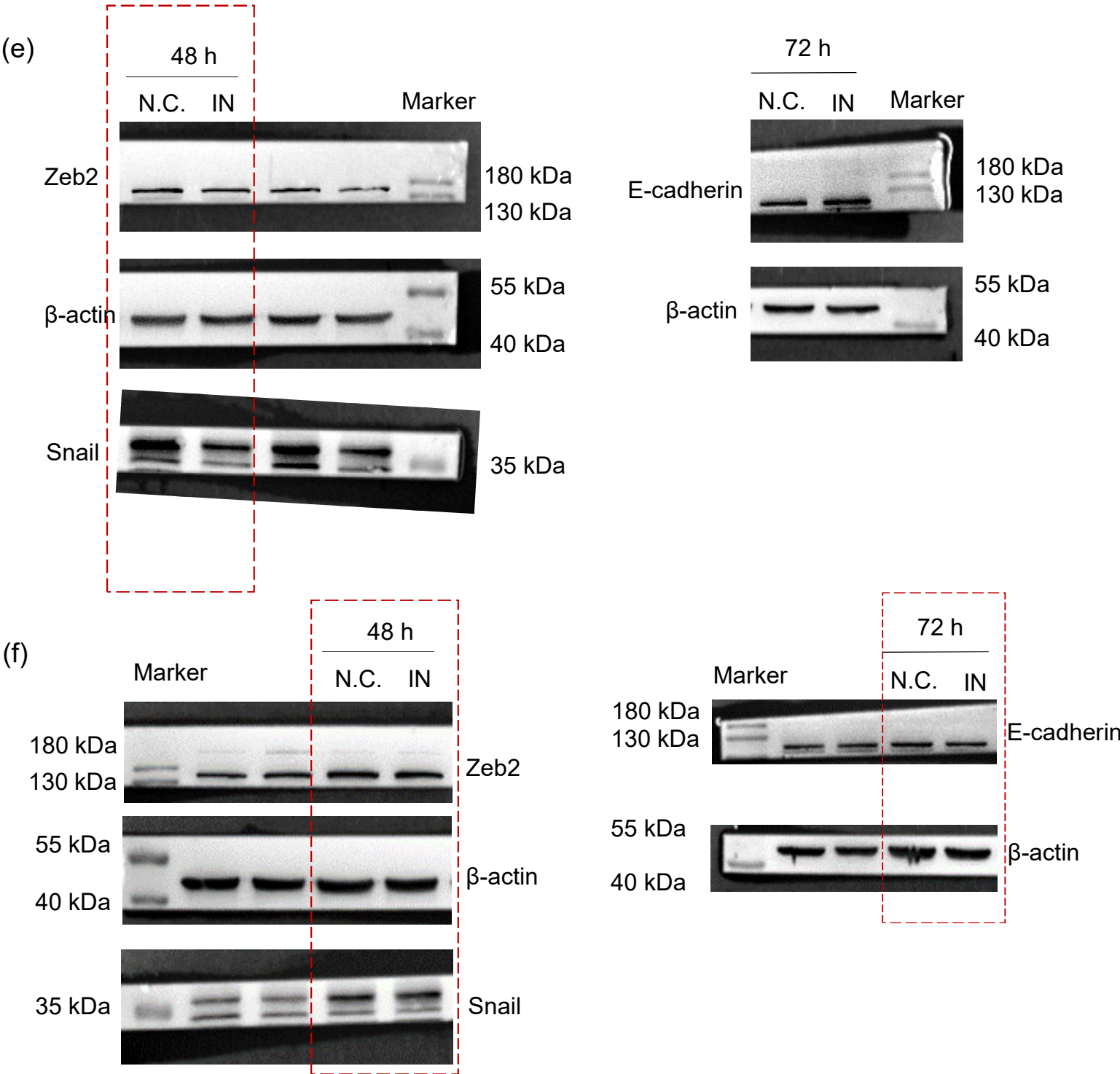

Supplement: Supplementary file 4 [file DataSheet_1.pdf]

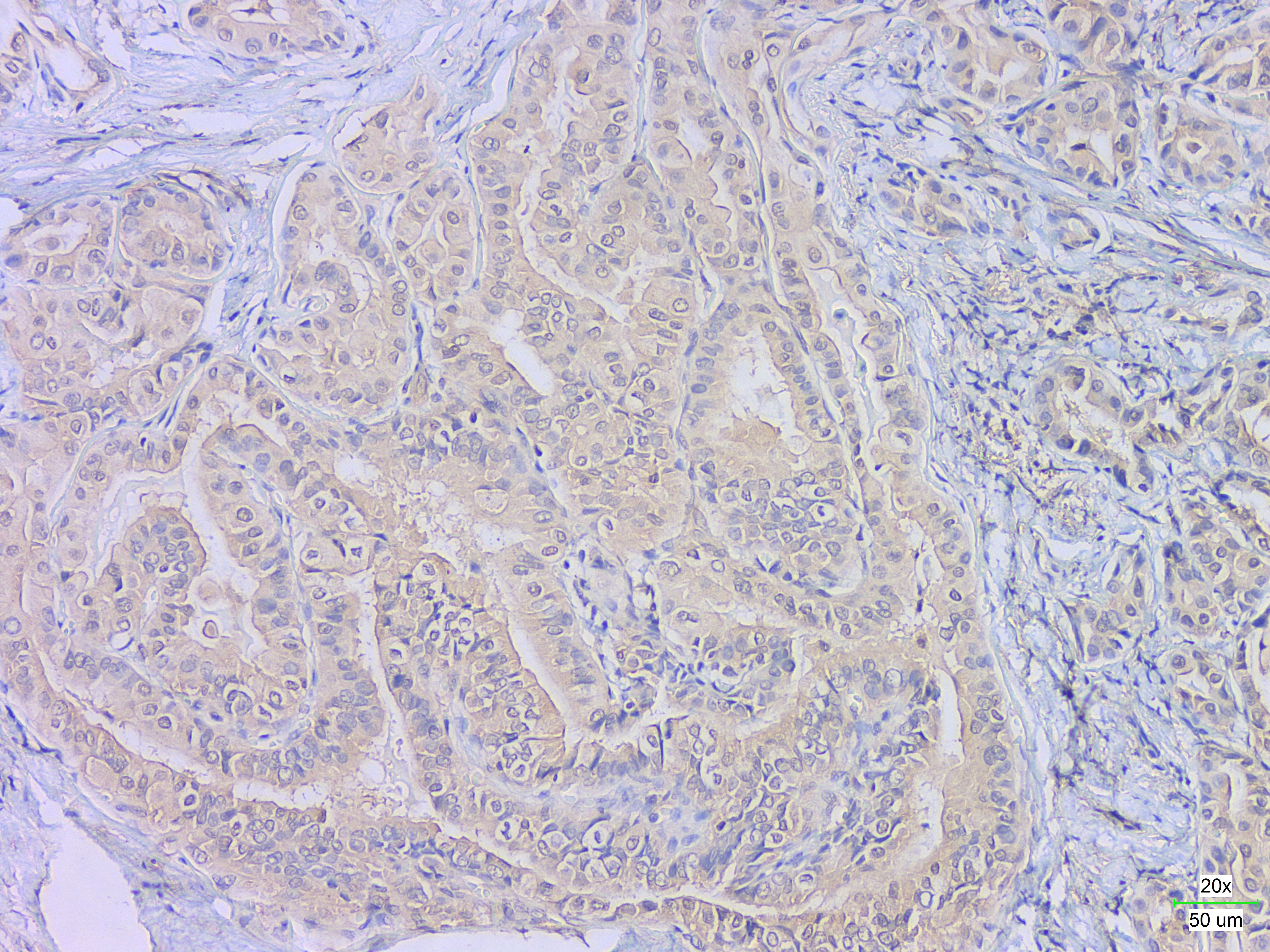

Supplement: Supplementary file 8 [file DataSheet_5.zip › In situ hybridization/B17-30444D2 hsa-mir-222-3p 200-1.jpg]

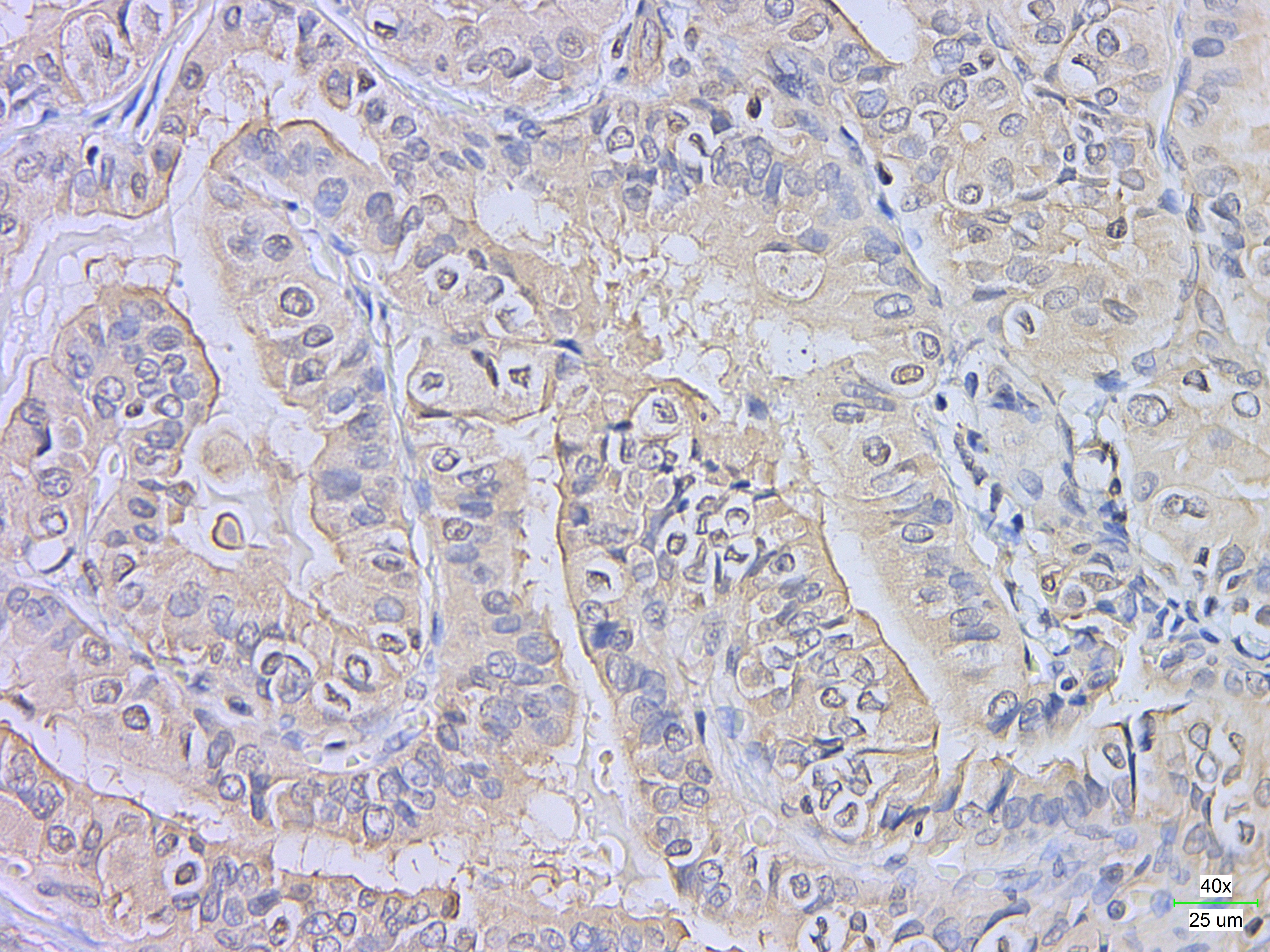

Supplement: Supplementary file 8 [file DataSheet_5.zip › In situ hybridization/B17-30444D2 hsa-mir-222-3p 400-3.jpg]

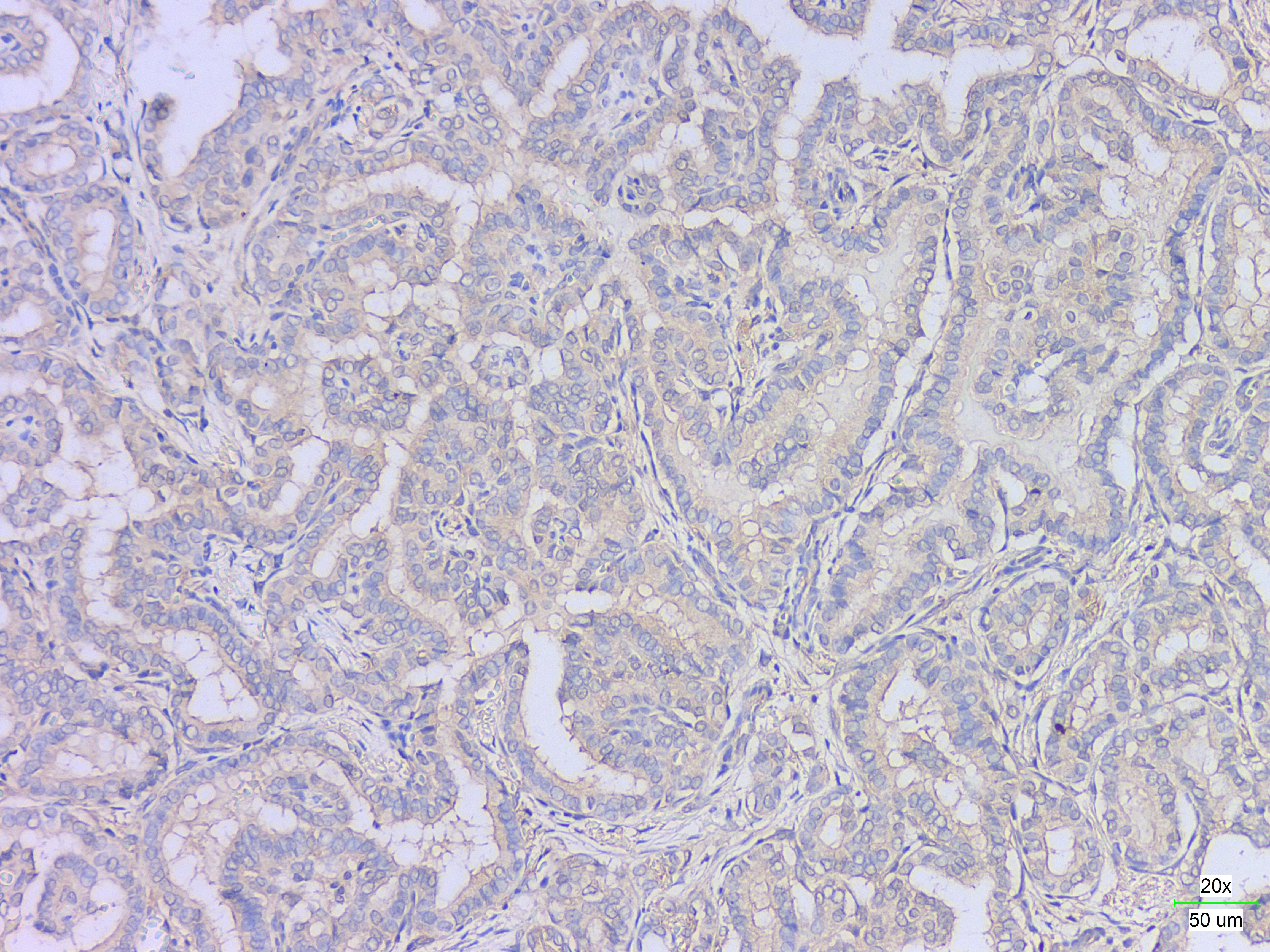

Supplement: Supplementary file 8 [file DataSheet_5.zip › In situ hybridization/B17-30866A2 hsa-mir-222-3p 200-3.jpg]

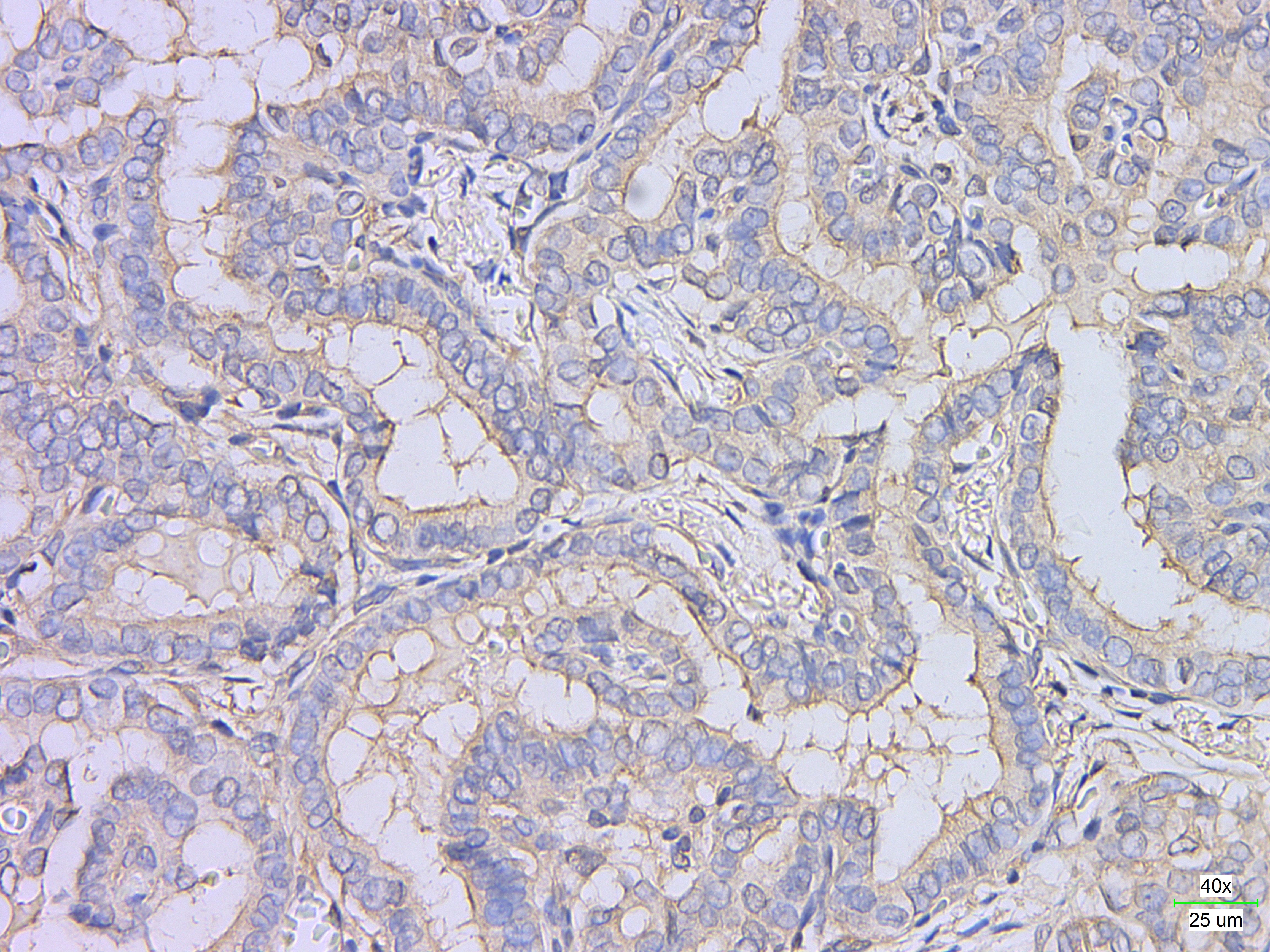

Supplement: Supplementary file 8 [file DataSheet_5.zip › In situ hybridization/B17-30866A2 hsa-mir-222-3p 400-2.jpg]

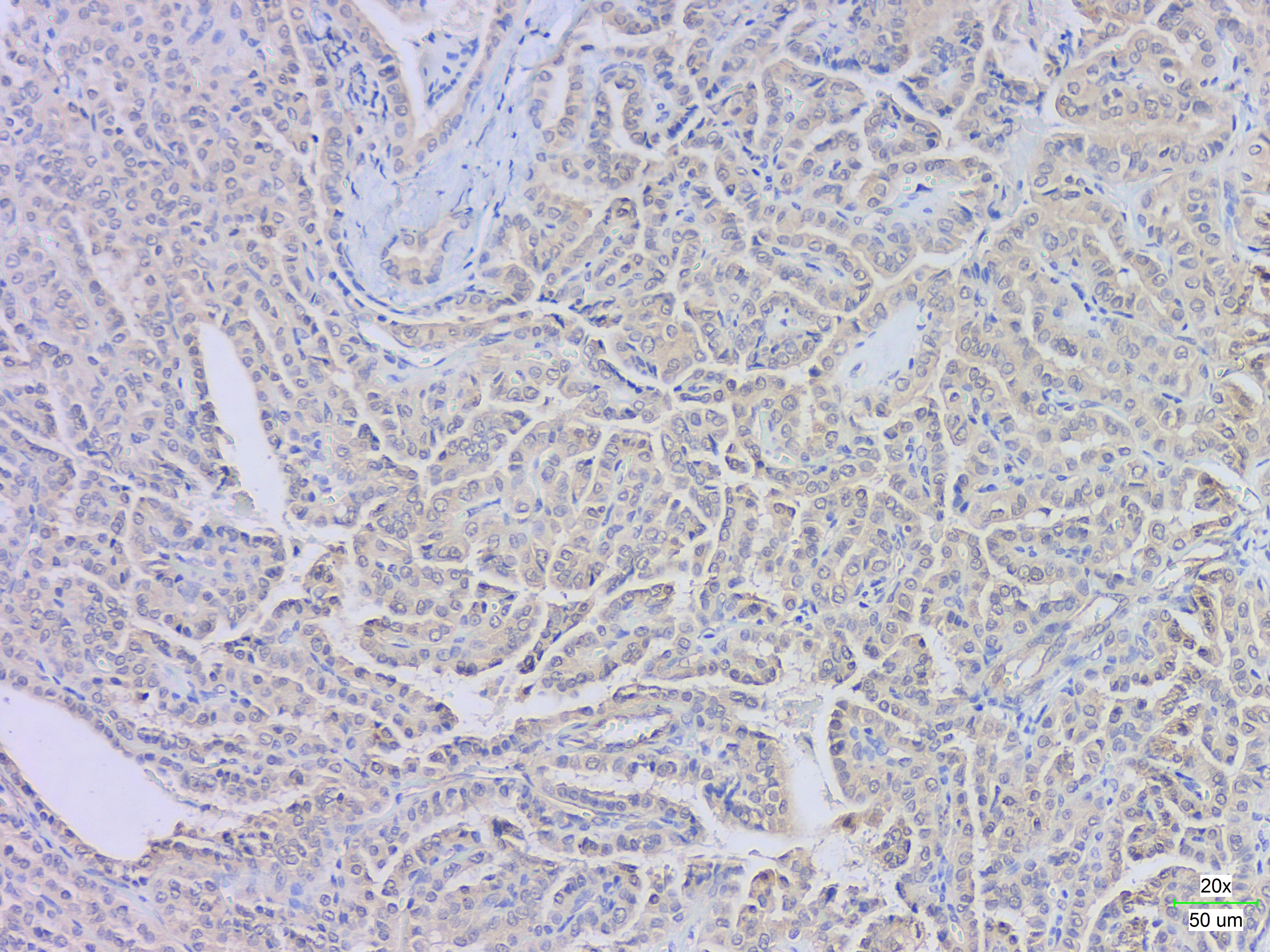

Supplement: Supplementary file 8 [file DataSheet_5.zip › In situ hybridization/B17-31172B2 hsa-mir-222-3p 200-3.jpg]

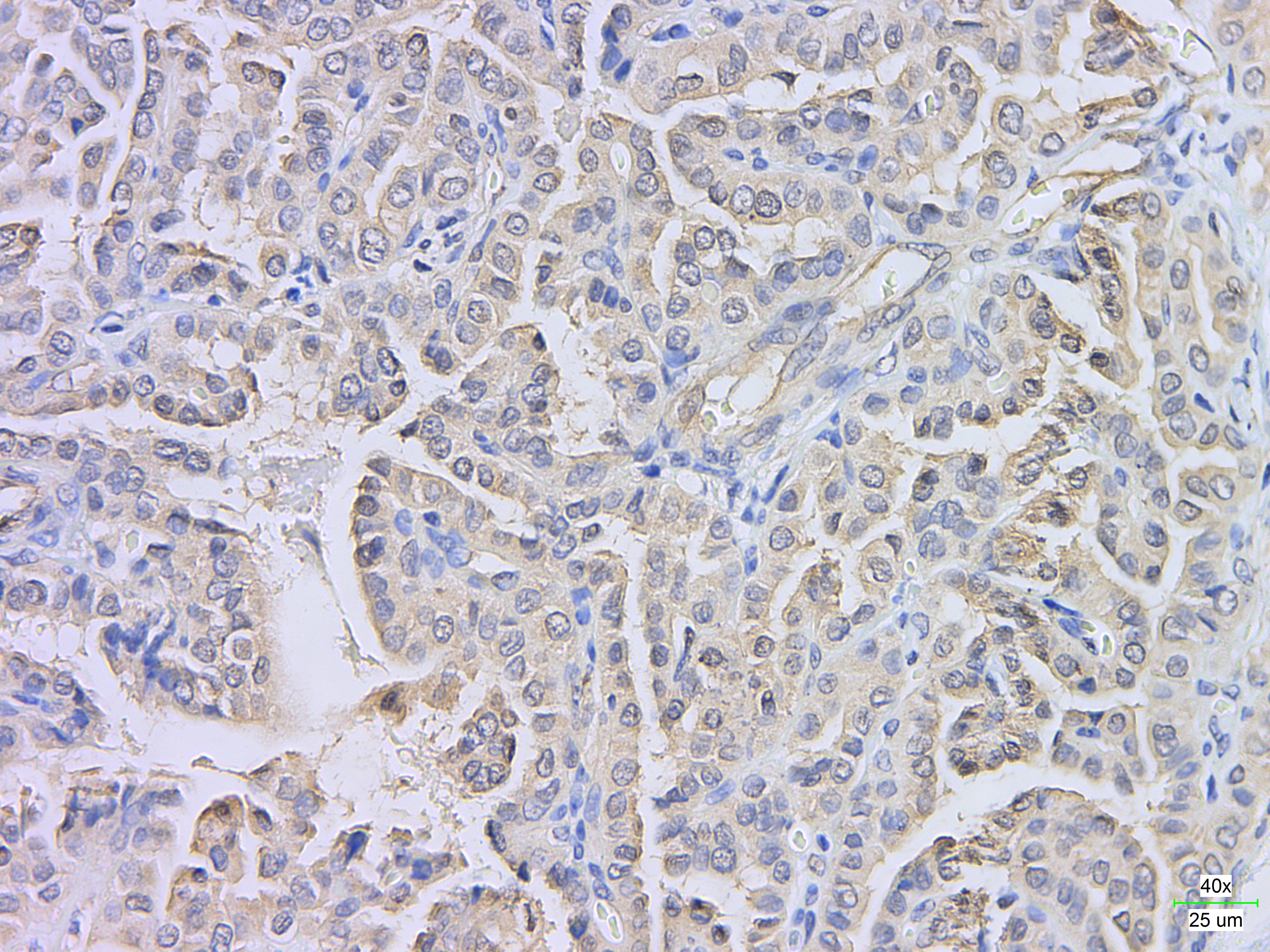

Supplement: Supplementary file 8 [file DataSheet_5.zip › In situ hybridization/B17-31172B2 hsa-mir-222-3p 400-1.jpg]

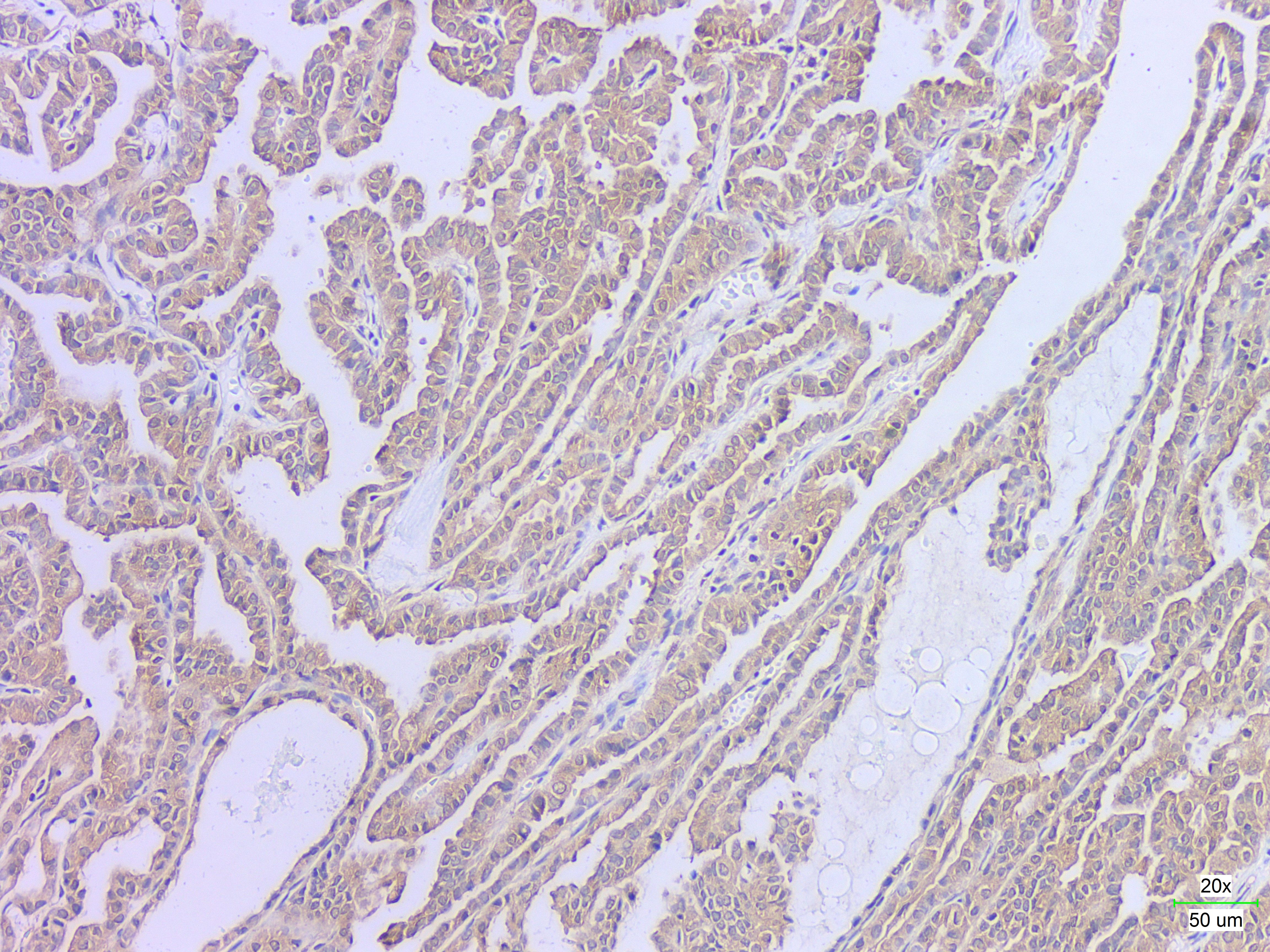

Supplement: Supplementary file 8 [file DataSheet_5.zip › In situ hybridization/B18-903B1 hsa-mir-222-3p 200-3.jpg]

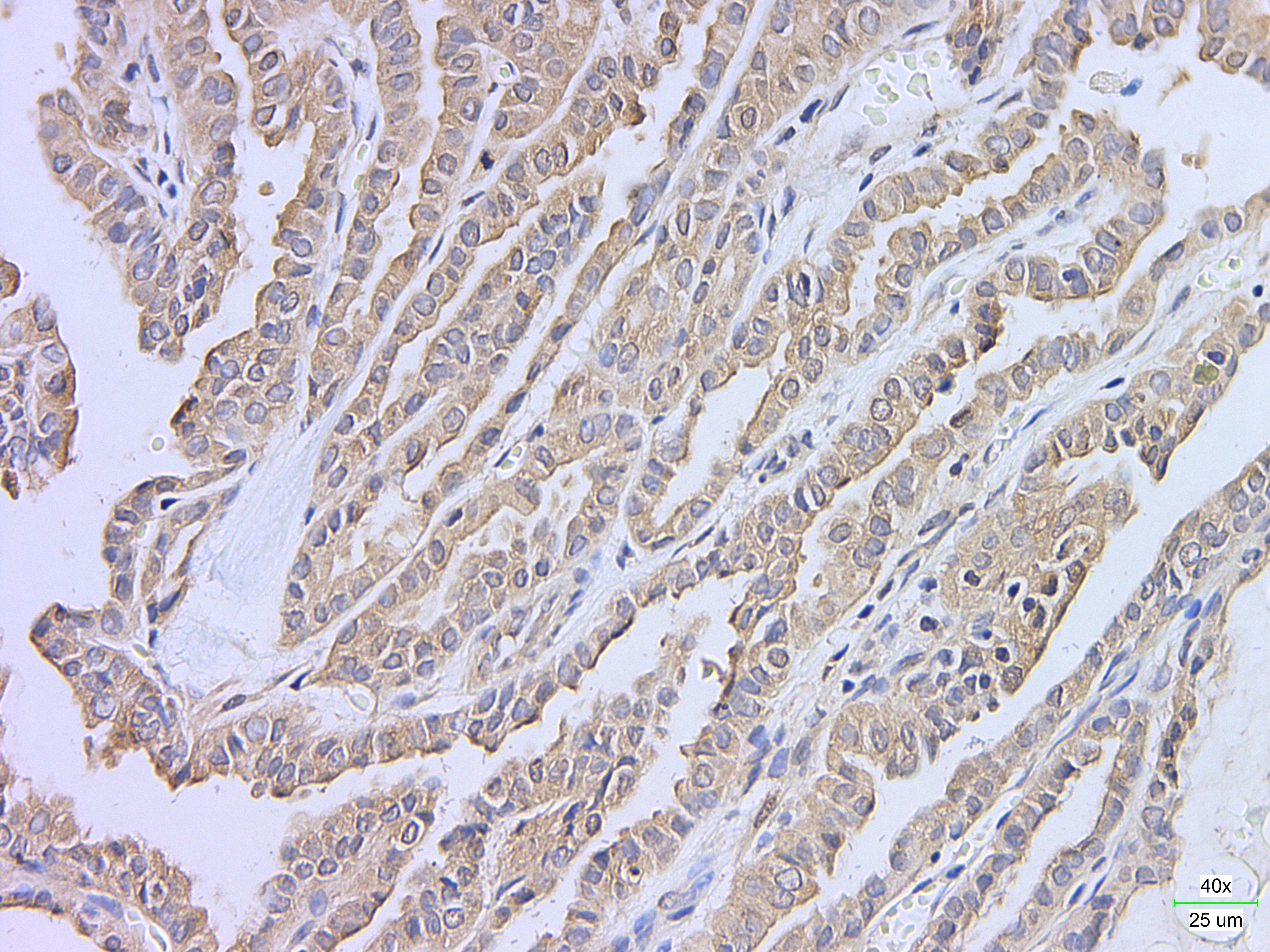

Supplement: Supplementary file 8 [file DataSheet_5.zip › In situ hybridization/B18-903B1 hsa-mir-222-3p 400-1.jpg]

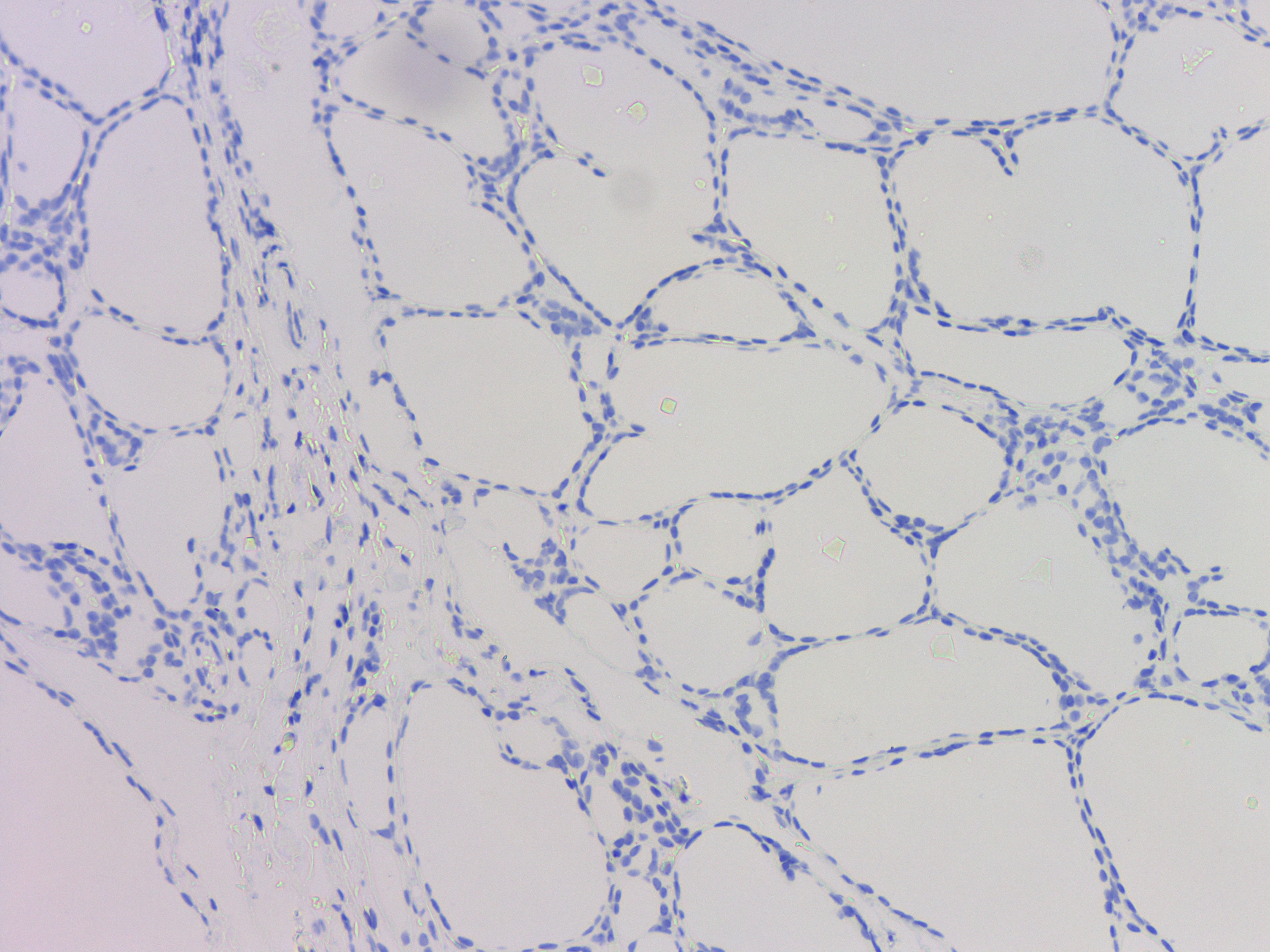

Supplement: Supplementary file 8 [file DataSheet_5.zip › In situ hybridization/B19-2236 B1 ╥⌡╢╘ 200-3.jpg]

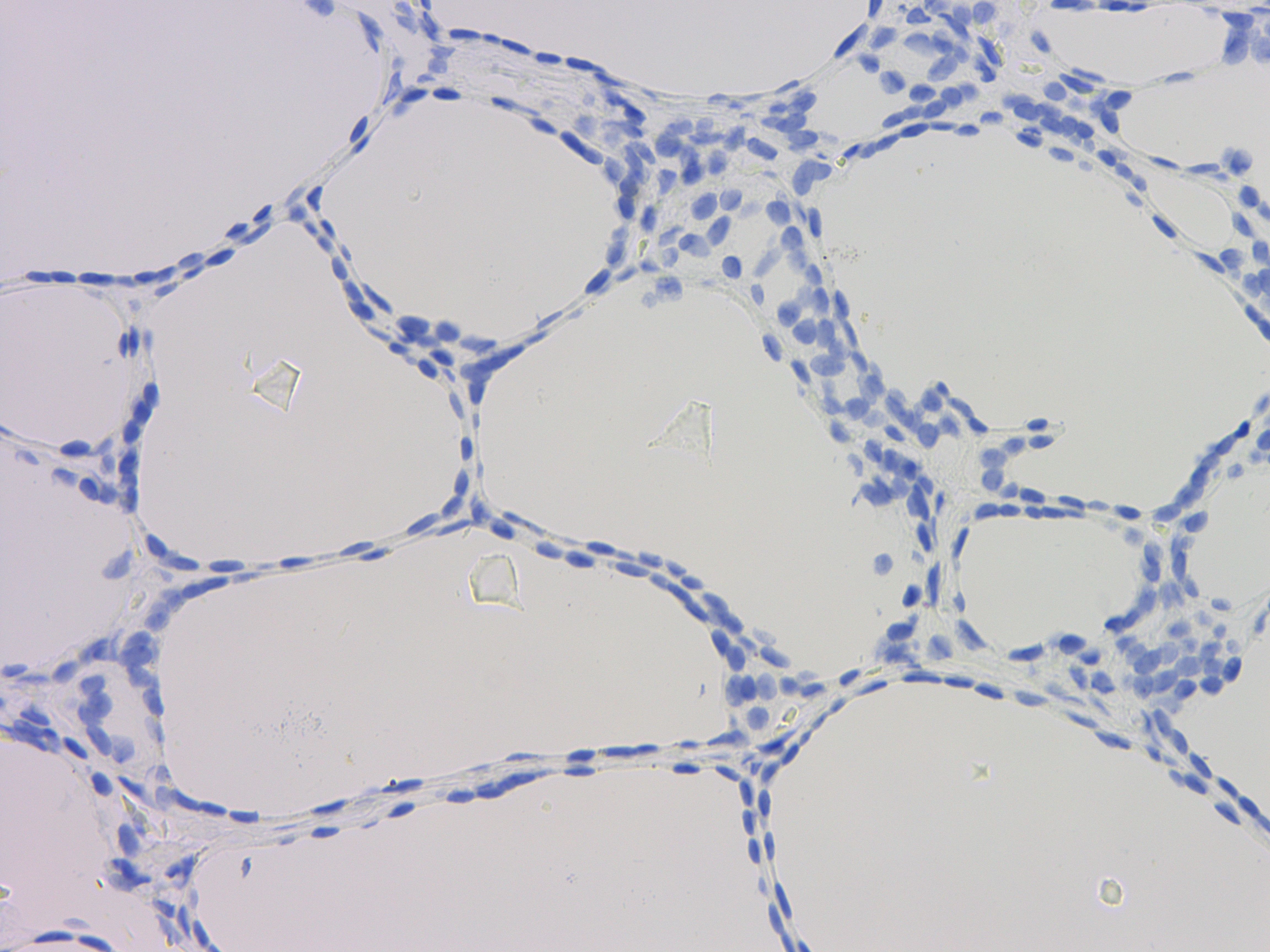

Supplement: Supplementary file 8 [file DataSheet_5.zip › In situ hybridization/B19-2236 B1 ╥⌡╢╘ 400-1.jpg]

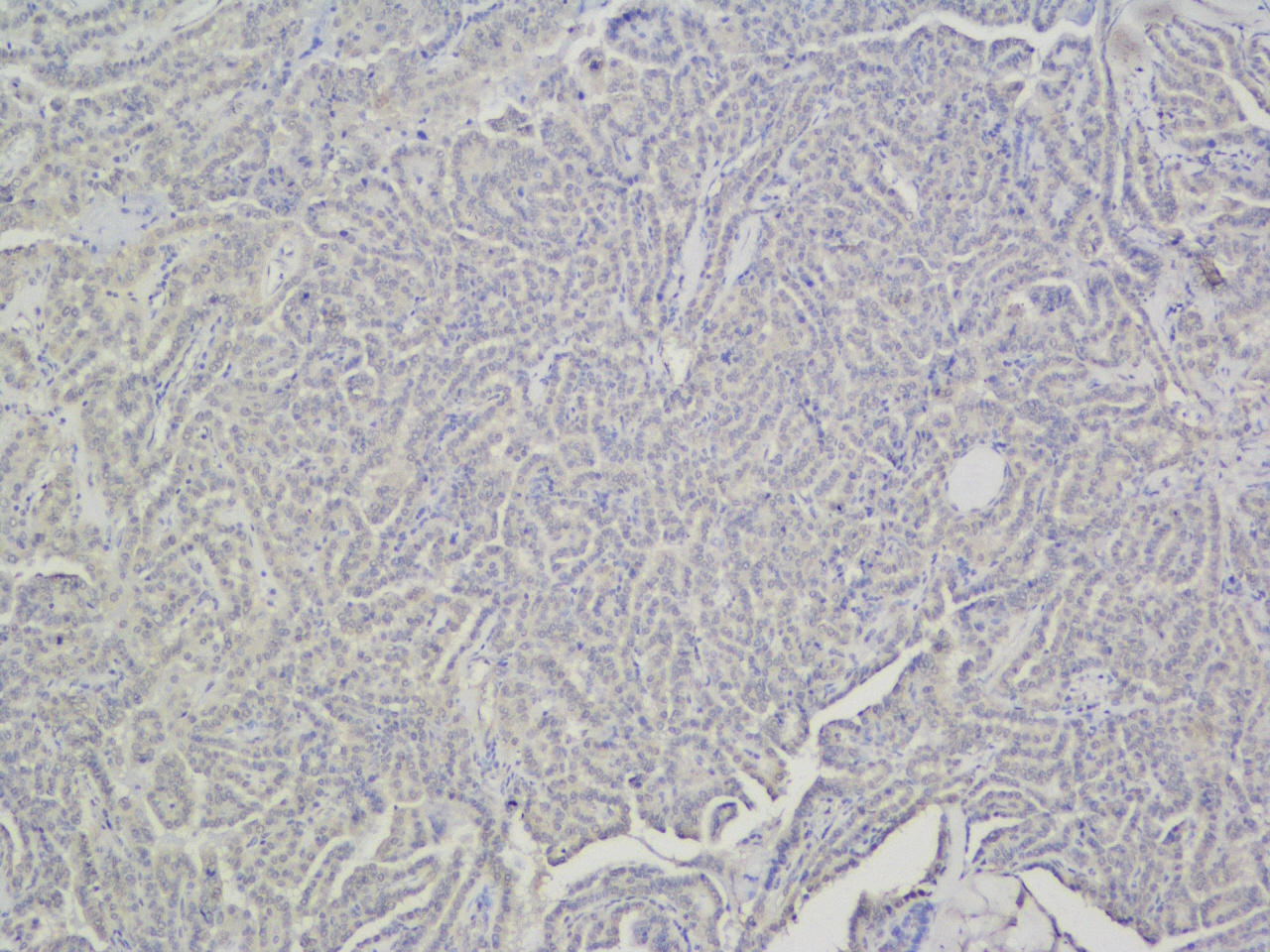

Supplement: Supplementary file 9 [file DataSheet_6.zip › immunohistochemistry/B17-31172B2 BP363ú¿1ú║100ú⌐ snail 100-1.jpg]

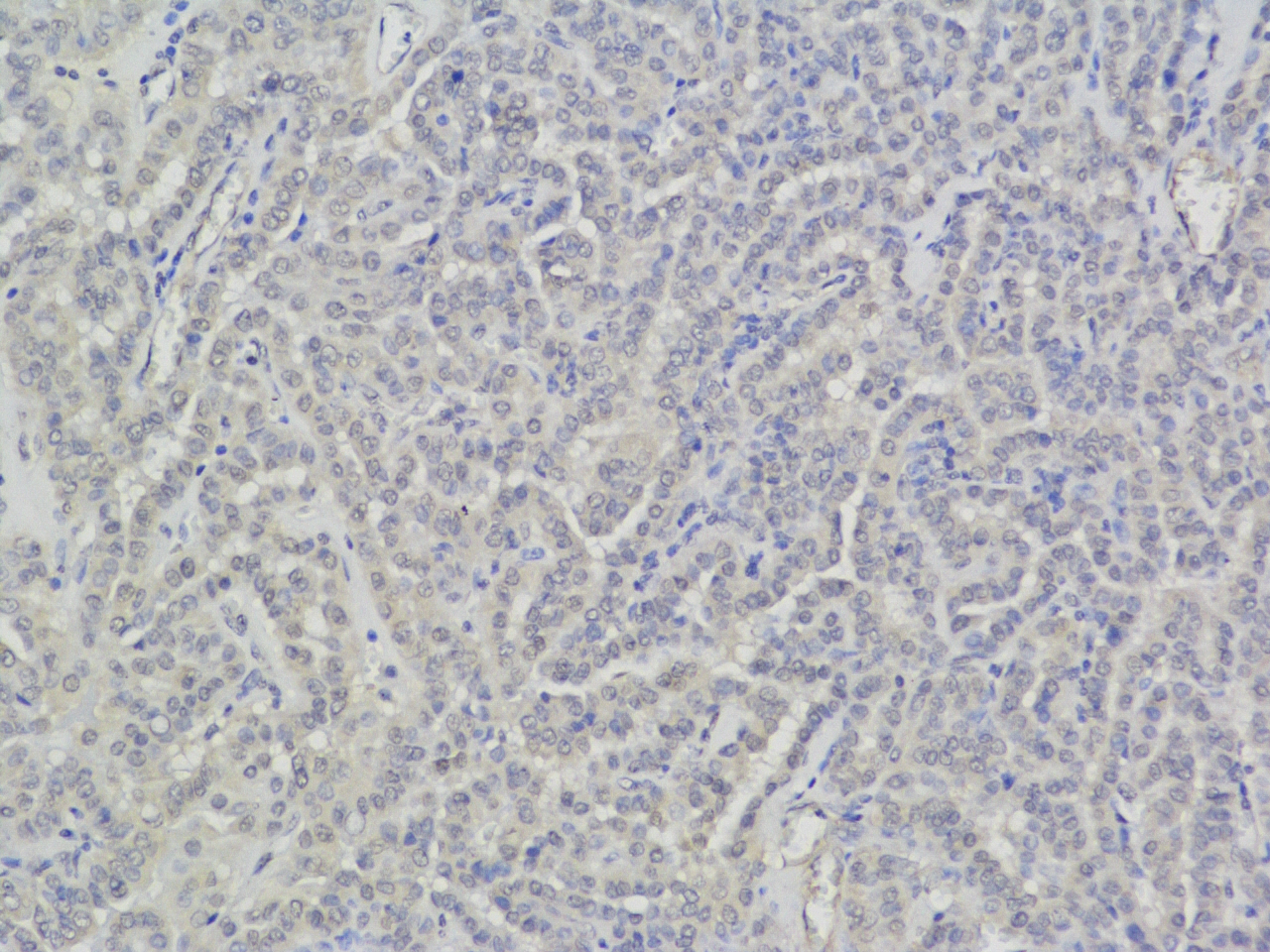

Supplement: Supplementary file 9 [file DataSheet_6.zip › immunohistochemistry/B17-31172B2 BP363ú¿1ú║100ú⌐ snail 200-1.jpg]

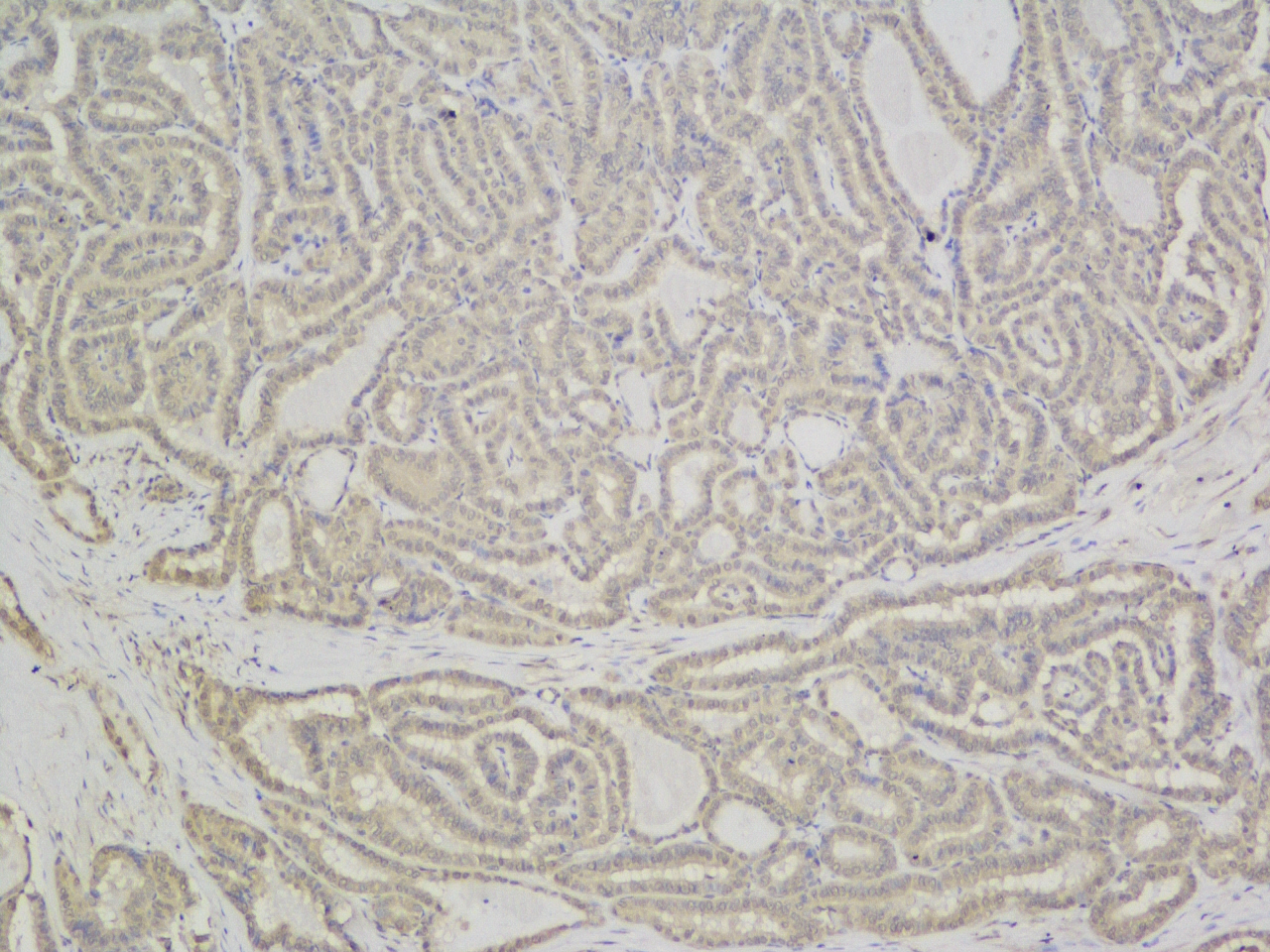

Supplement: Supplementary file 9 [file DataSheet_6.zip › immunohistochemistry/B17-31411C2 BP363ú¿1ú║100ú⌐ snail 100-1.jpg]

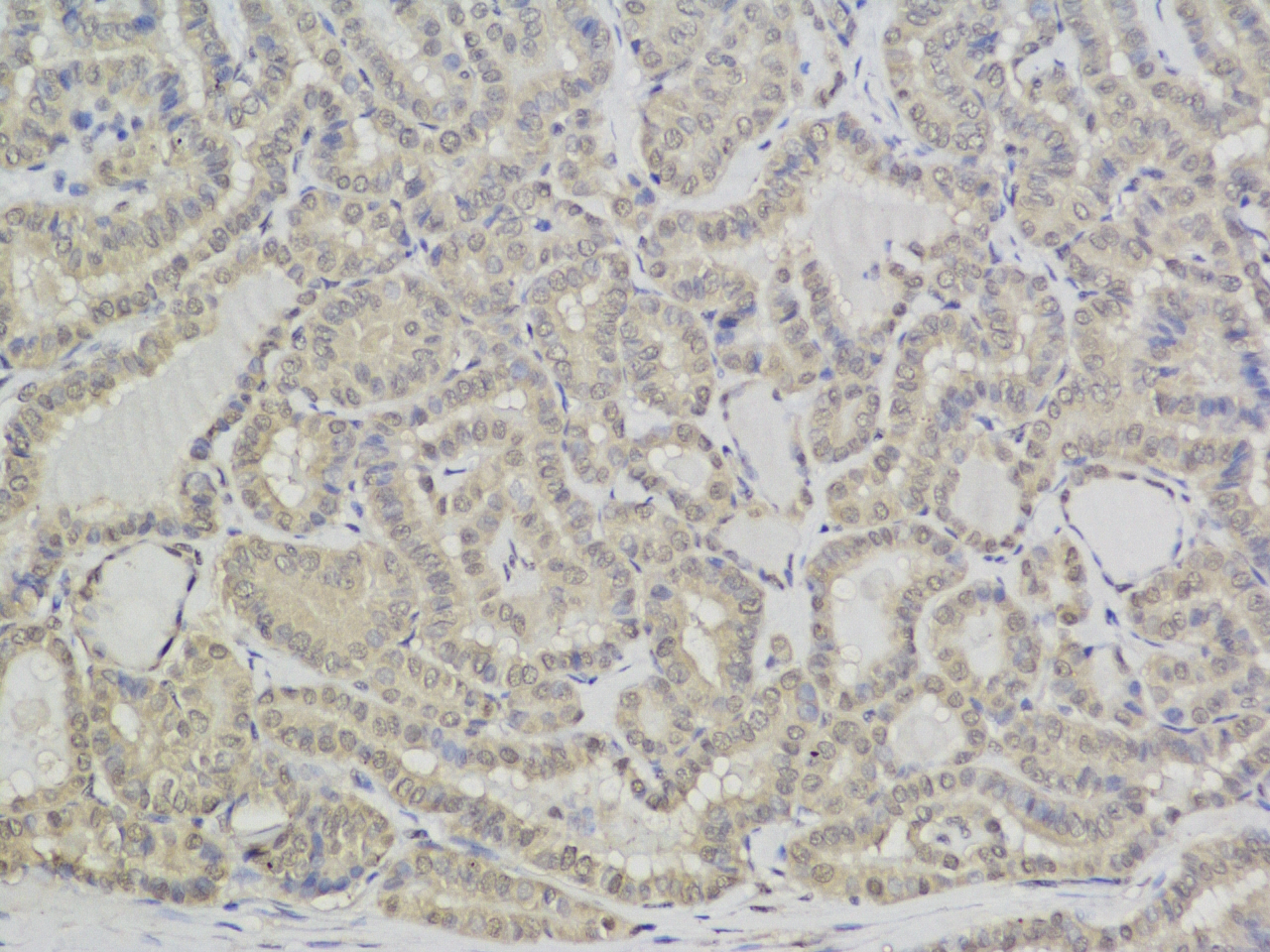

Supplement: Supplementary file 9 [file DataSheet_6.zip › immunohistochemistry/B17-31411C2 BP363ú¿1ú║100ú⌐ snail 200-1.jpg]
