# Supplementary material for: BRAFV600E Mutation-Responsive miRNA-222-3p Promotes Metastasis of Papillary Thyroid Cancer Cells via Snail-Induced EMT
Source: Front Endocrinol (Lausanne). 2022 May 16;13:843334. doi: 10.3389/fendo.2022.843334 (PMC9148970; doi:10.3389/fendo.2022.843334)

B-CPAP IN 1

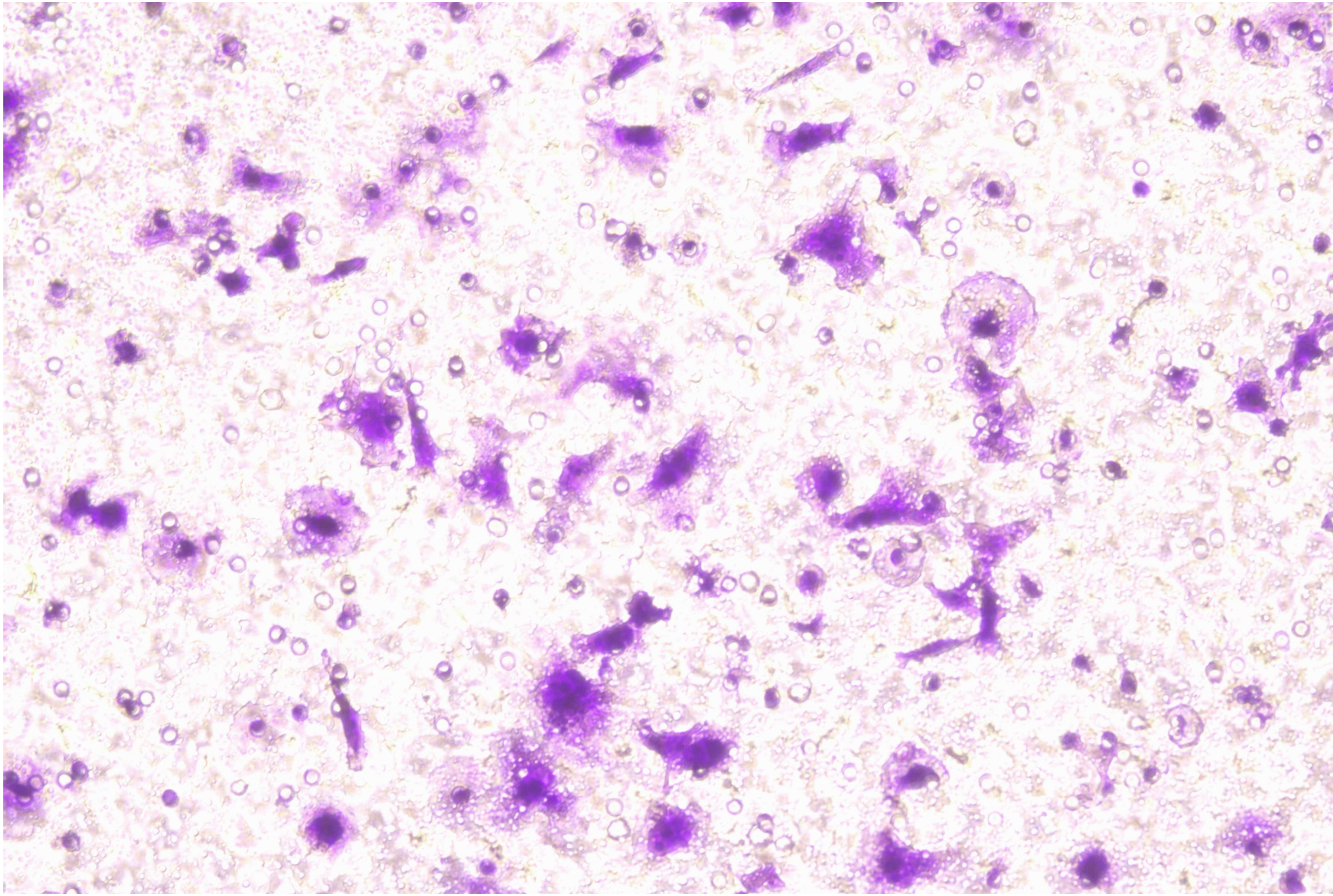

B-CPAP IN 2

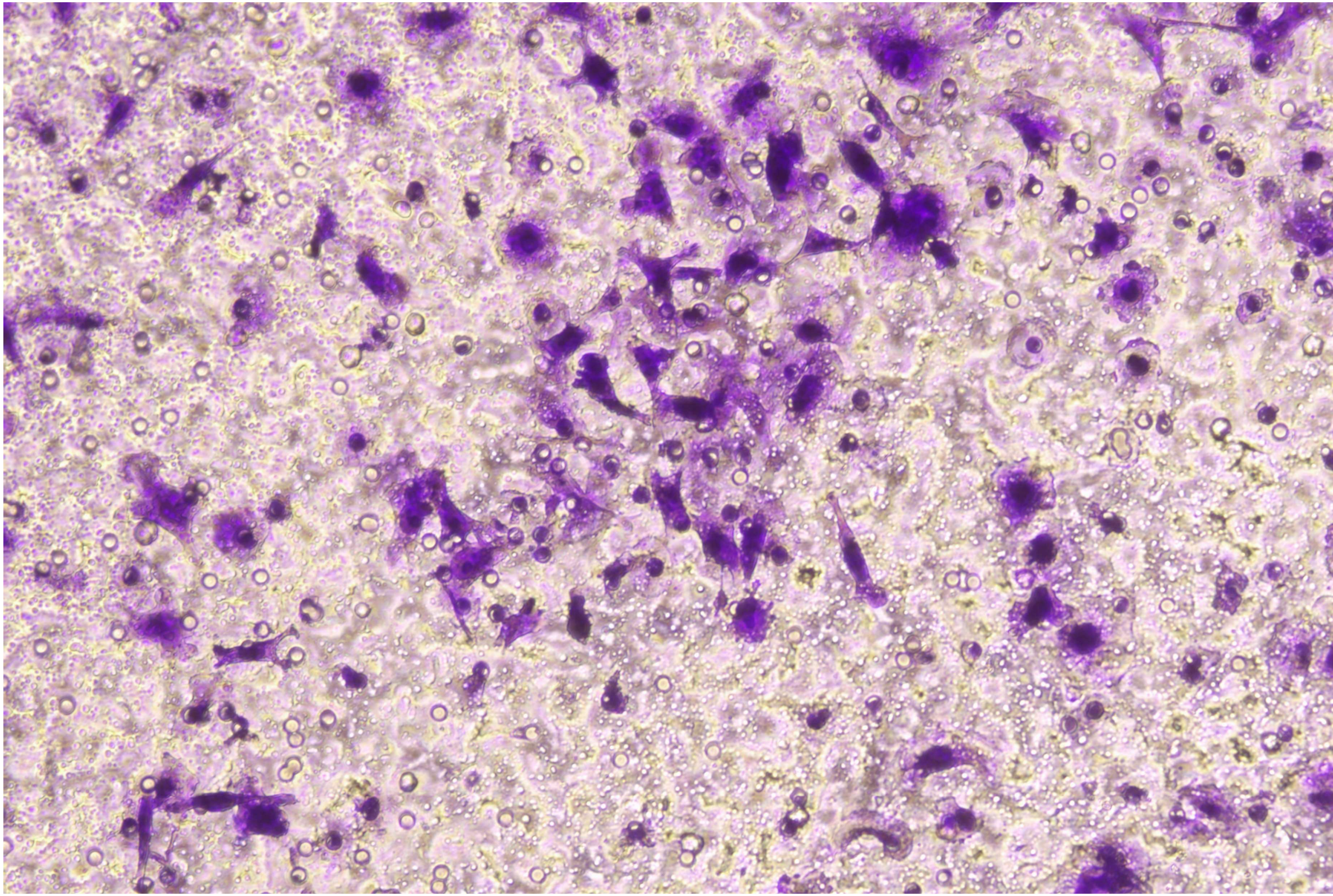

B-CPAP IN 3

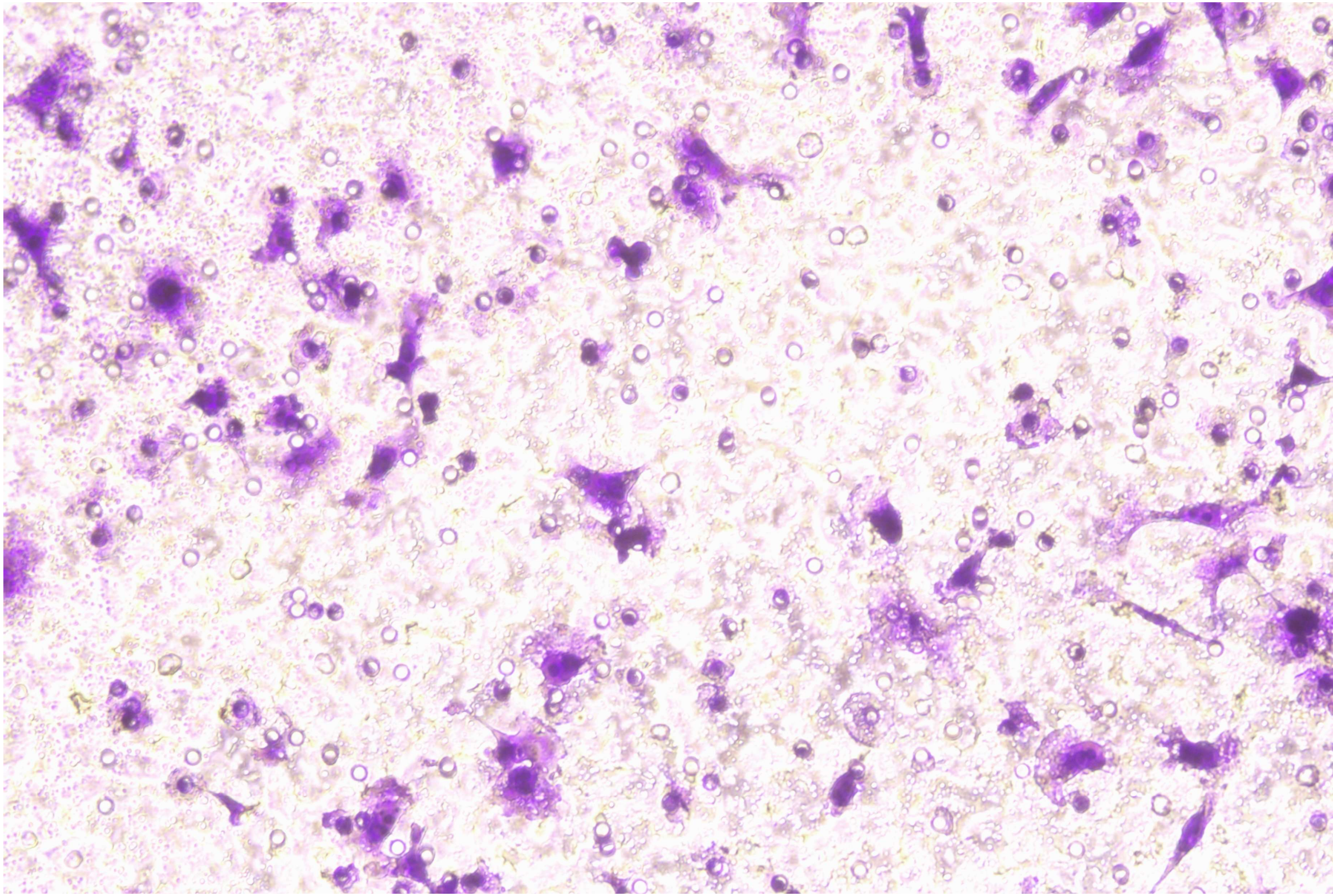

B-CPAP IN 4

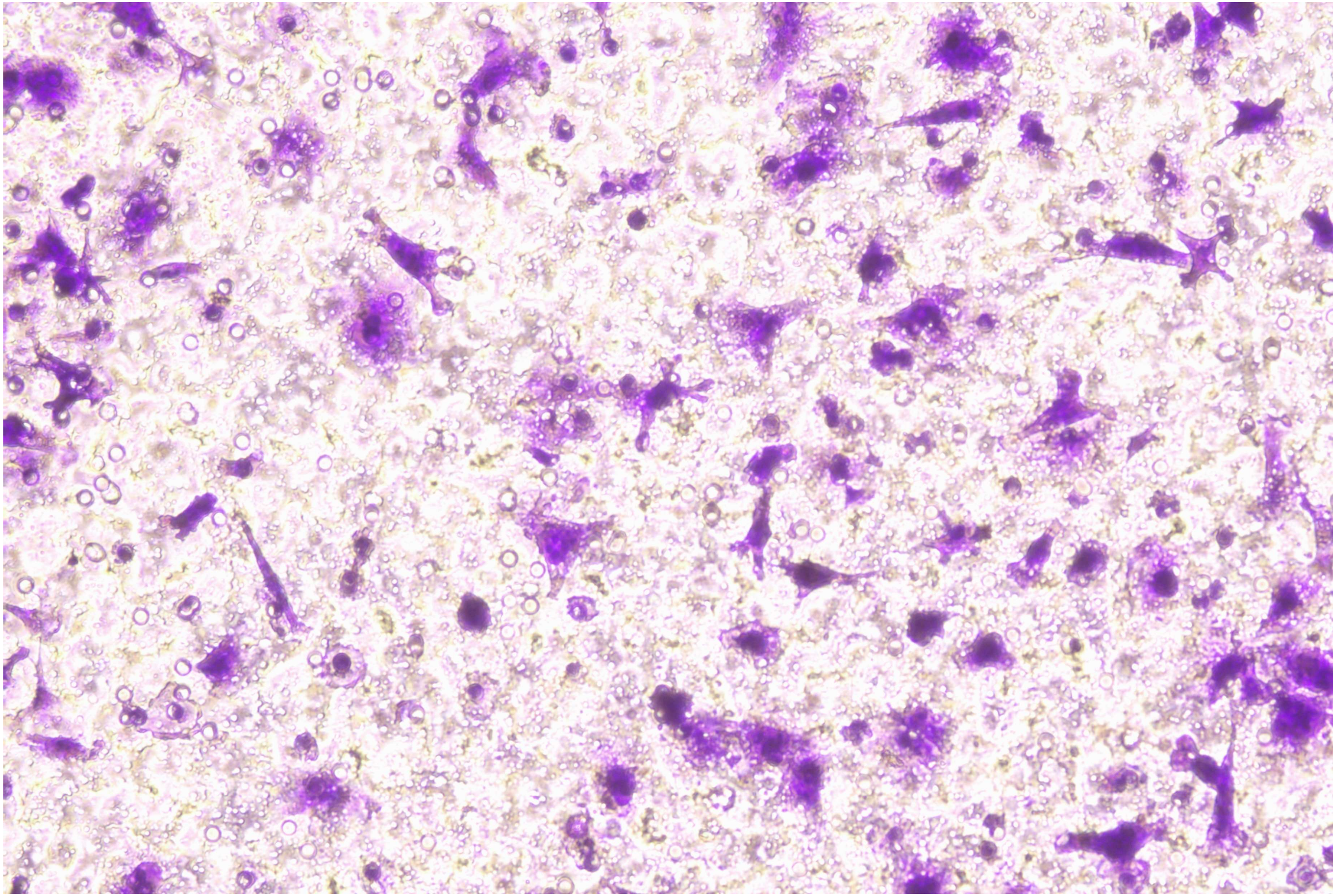

B-CPAP IN 5

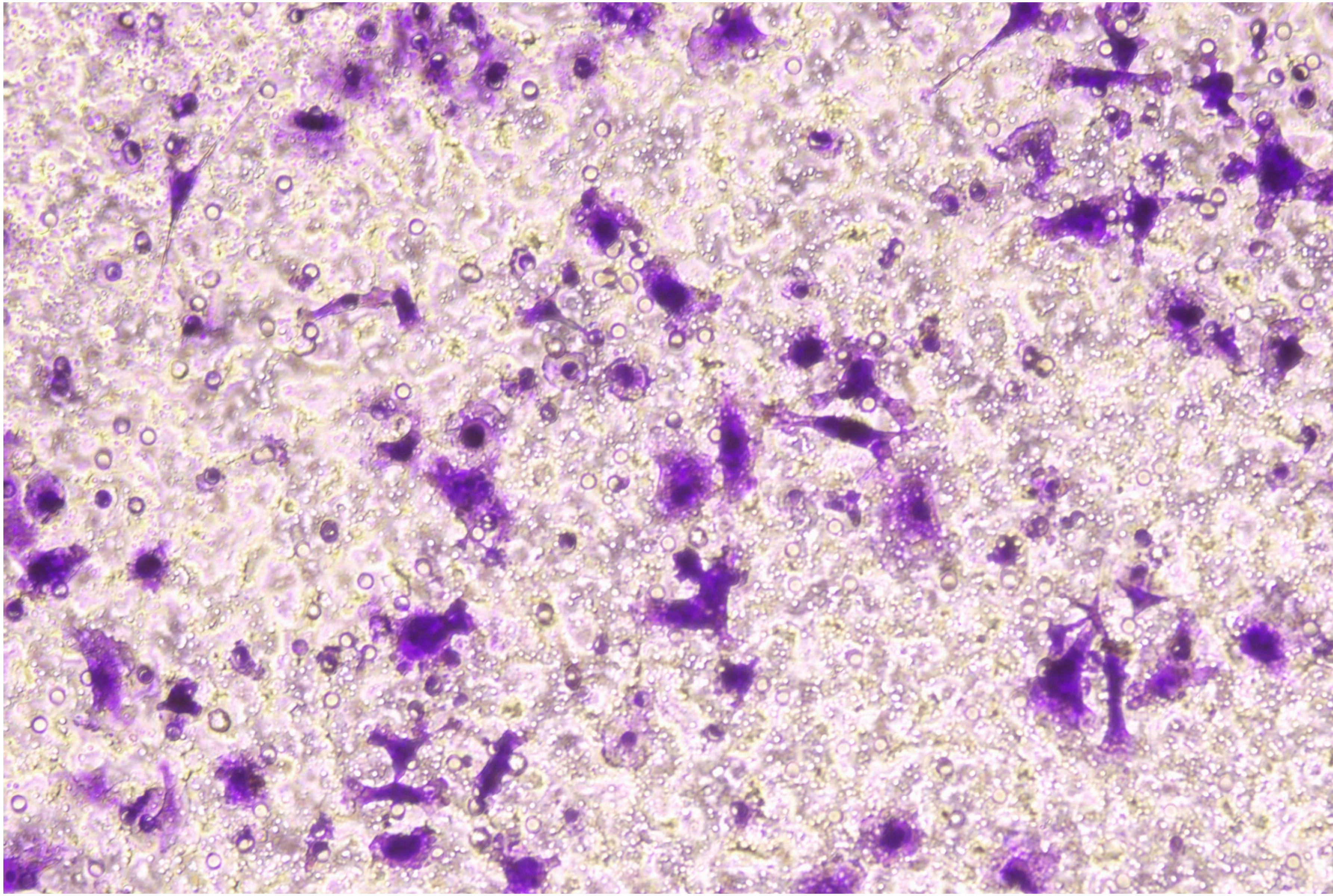

B-CPAP NC 1

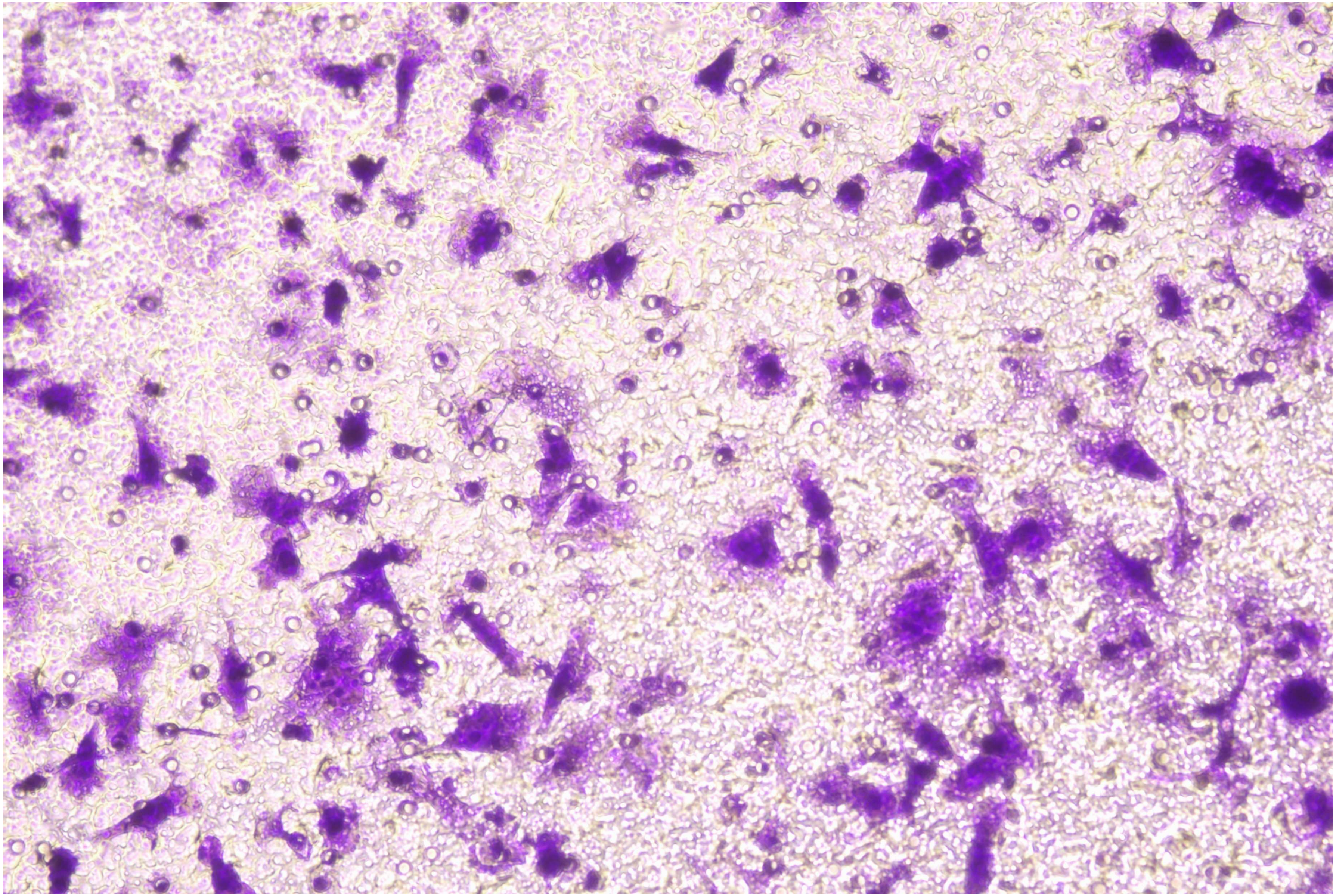

B-CPAP NC 2

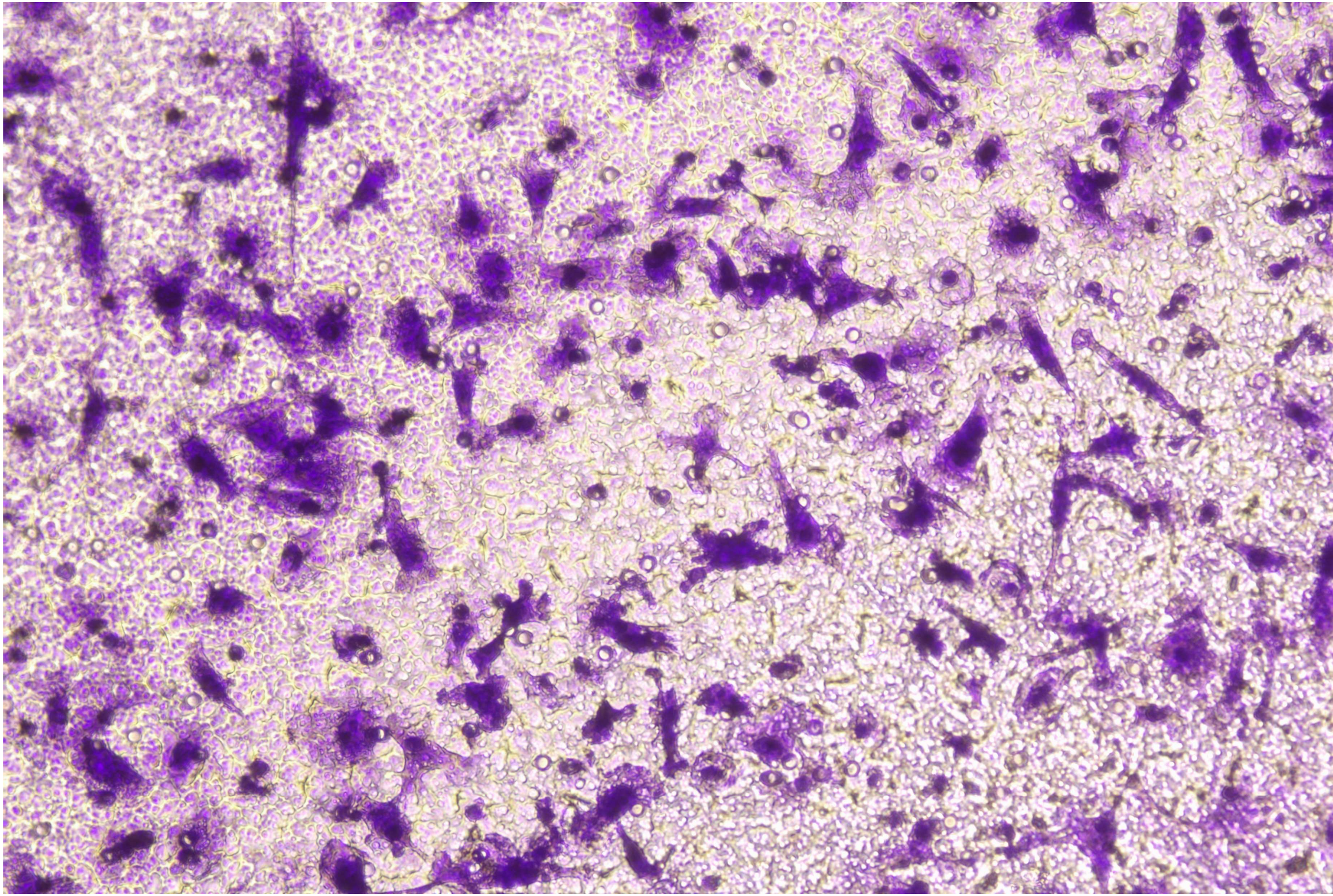

B-CPAP NC 3

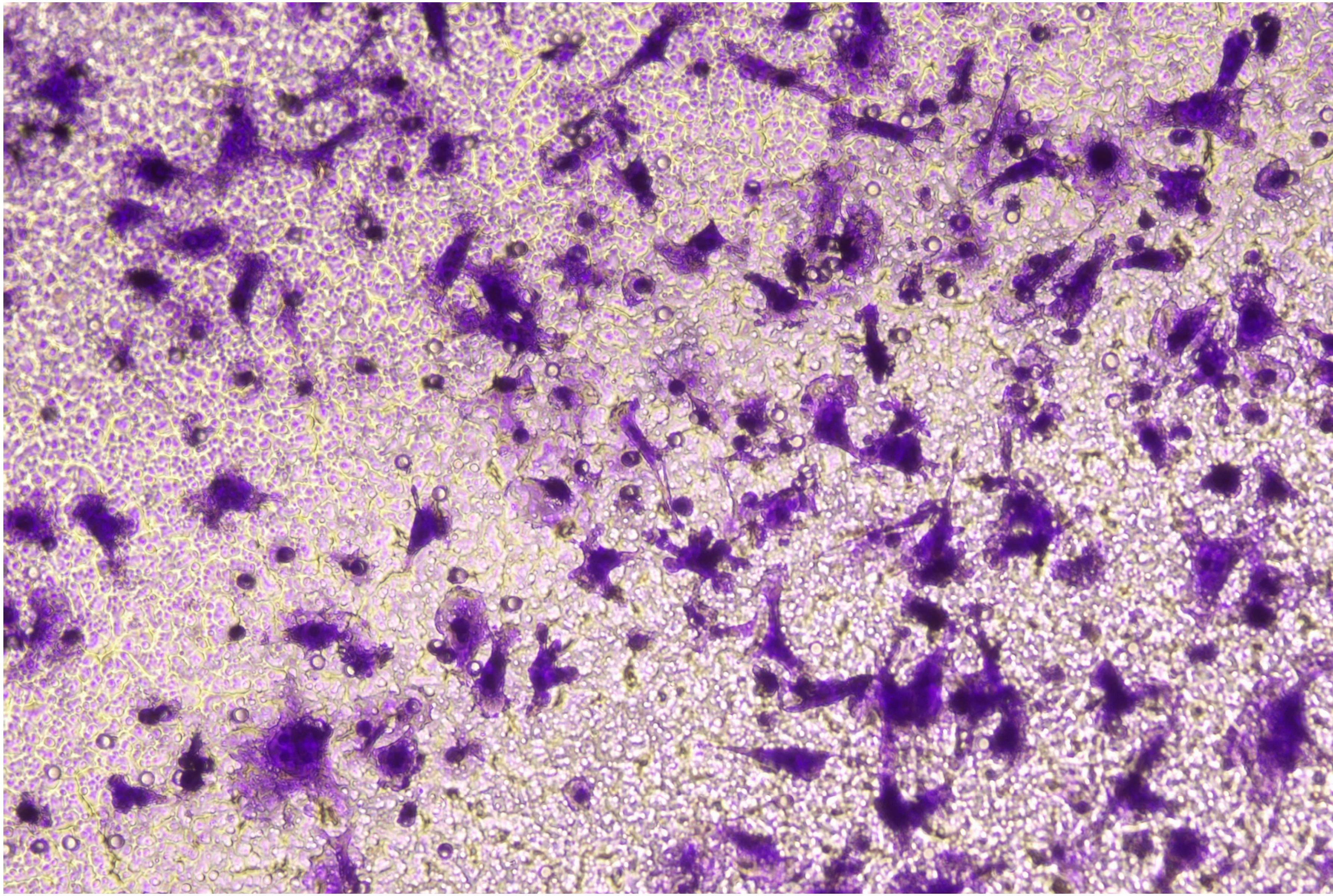

B-CPAP NC 4

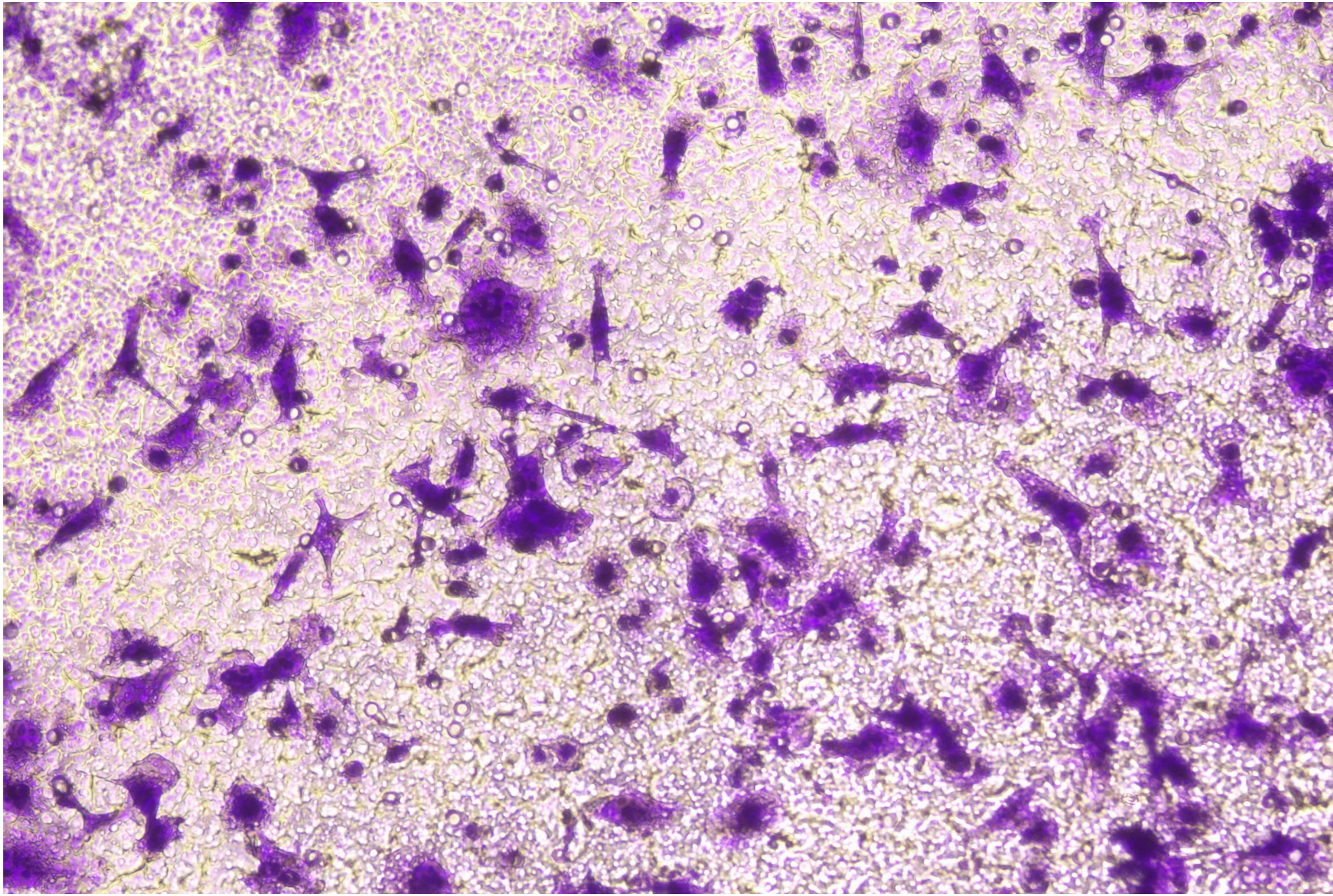

B-CPAP NC 5

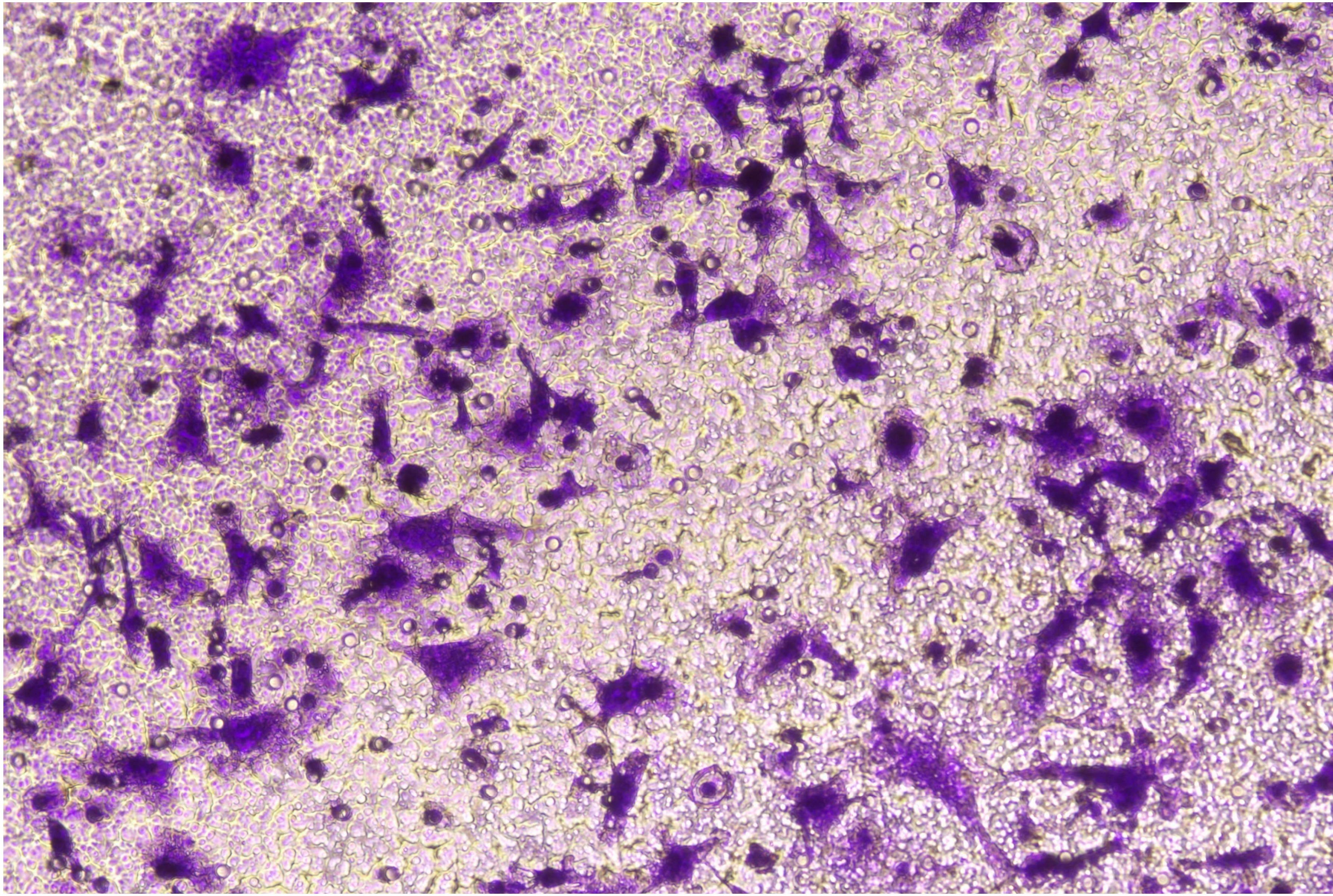

TPC-1 IN 1

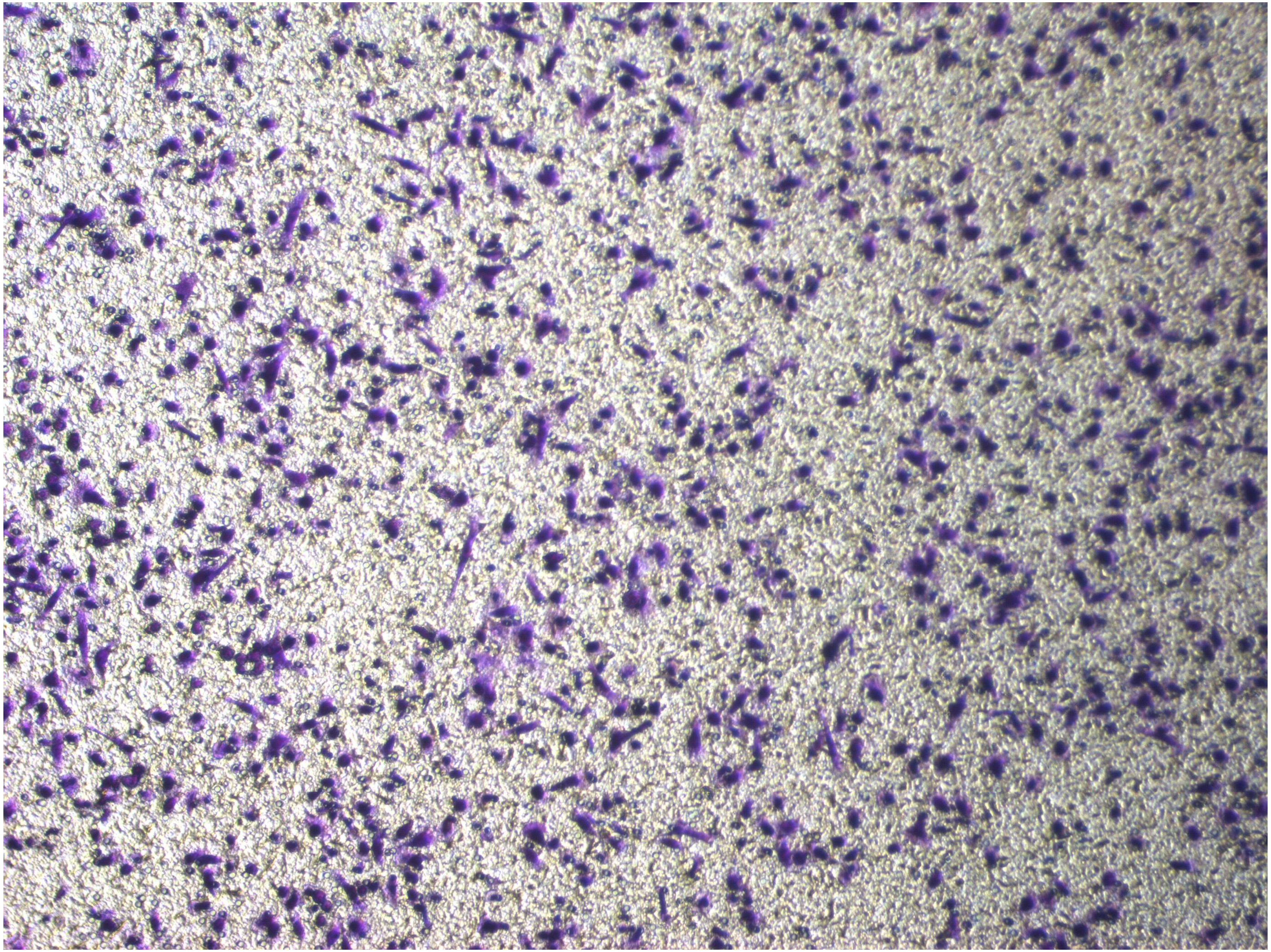

TPC-1 IN 2

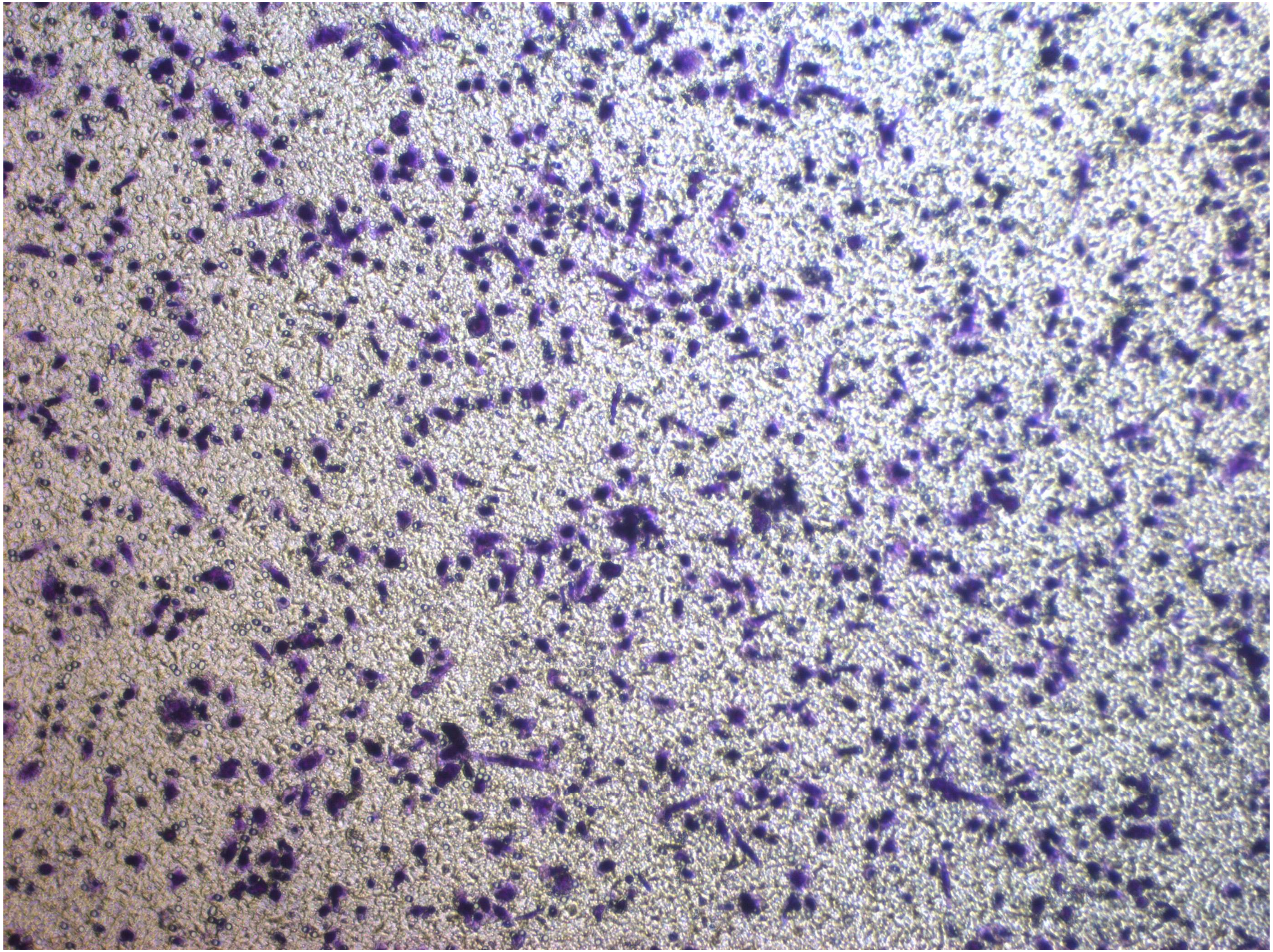

TPC-1 IN 3

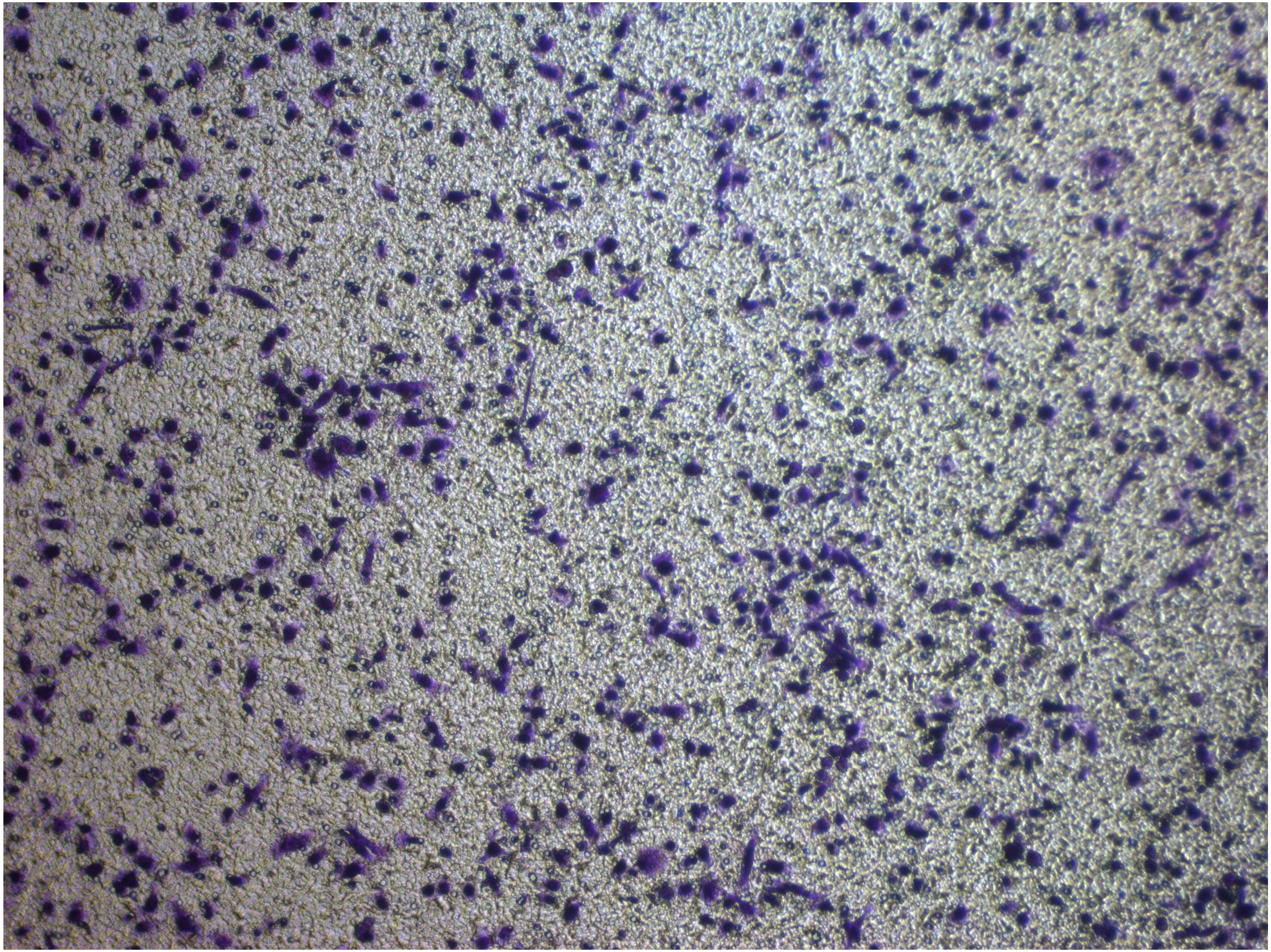

TPC-1 NC 1

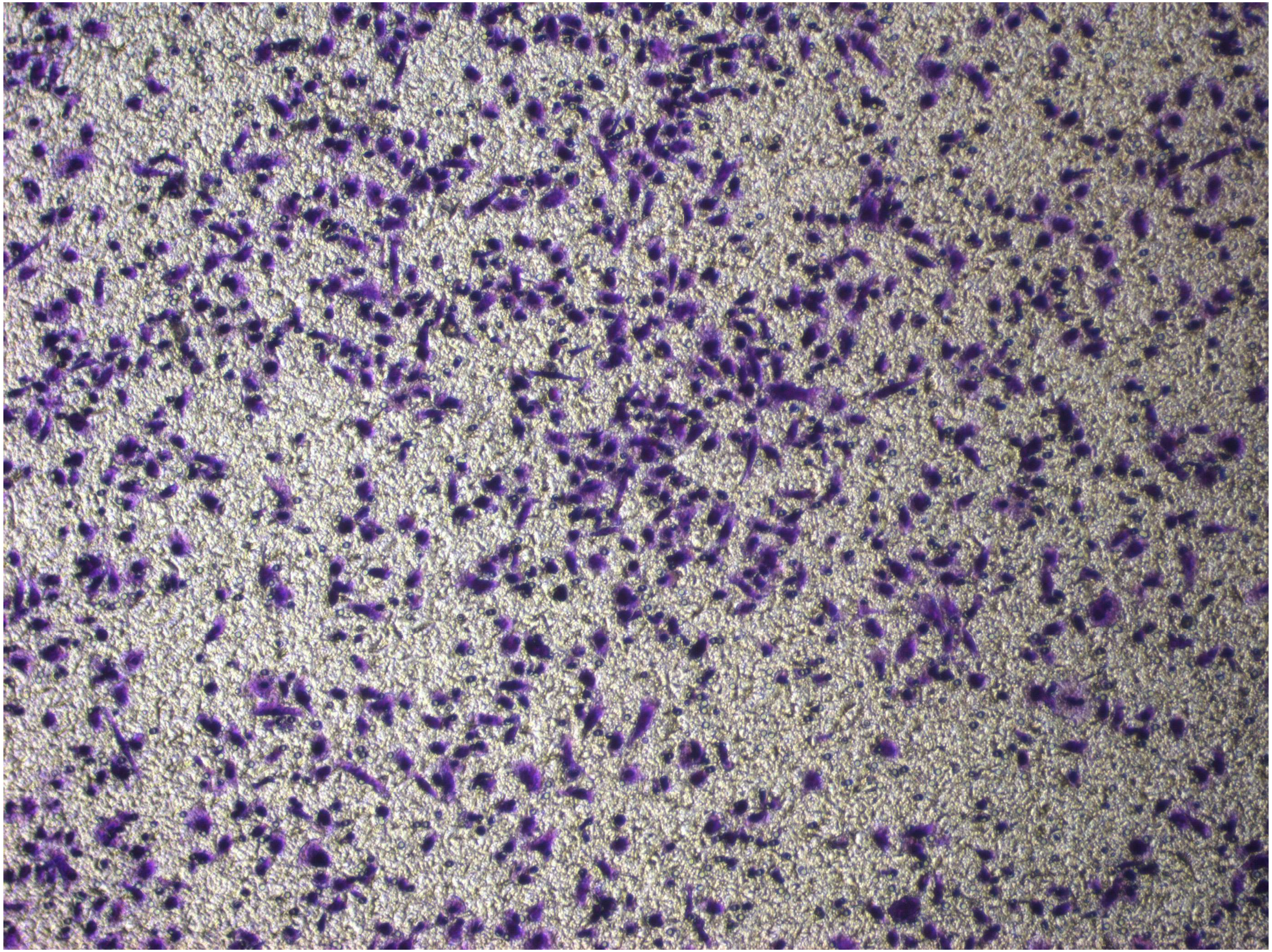

TPC-1 NC 2

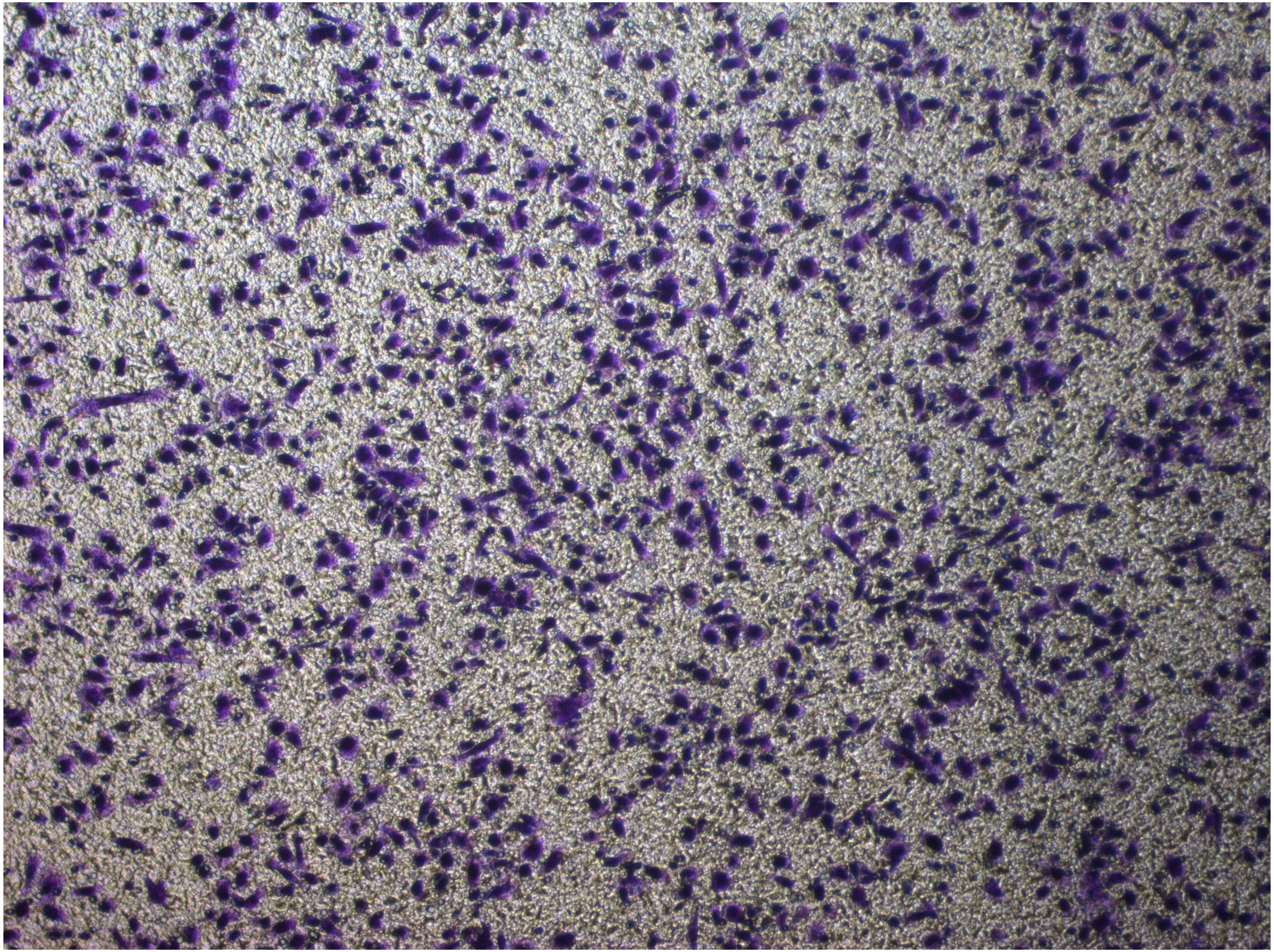

TPC-1 NC 3

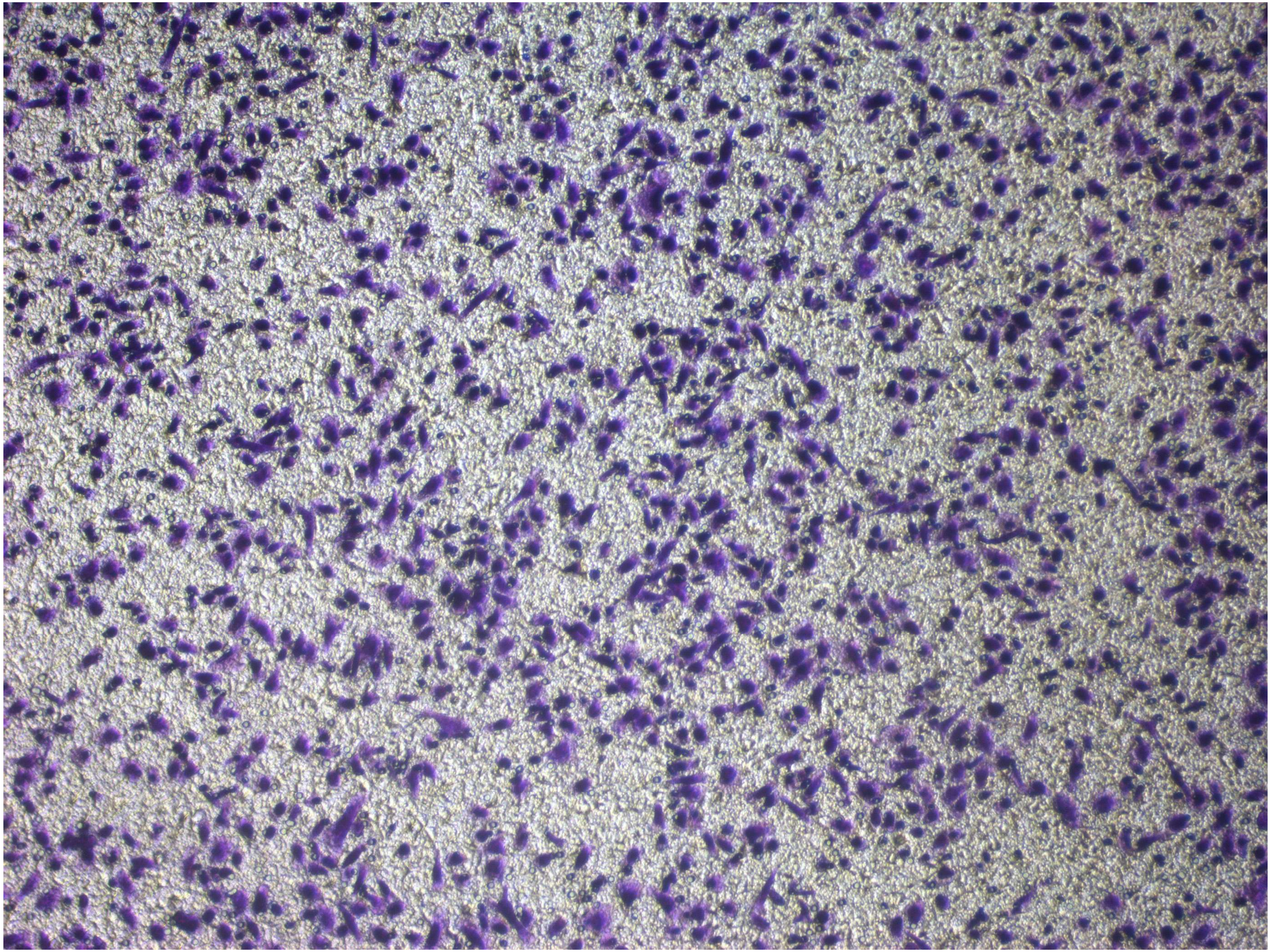

Supplement: Supplementary file 5 [file DataSheet_2.pdf]
