# Supplementary material for: BRAFV600E Mutation-Responsive miRNA-222-3p Promotes Metastasis of Papillary Thyroid Cancer Cells via Snail-Induced EMT
Source: Front Endocrinol (Lausanne). 2022 May 16;13:843334. doi: 10.3389/fendo.2022.843334 (PMC9148970; doi:10.3389/fendo.2022.843334)
Supplement: Supplementary file 6 [file DataSheet_3.zip › TCGA/Supplementary material.pdf]

## Supplementary Material

## UCSC xena

UCSC Xena (http://xena.ucsc.edu/) is a genome-related database including many tumor research database functions, providing visual analysis for public data centers. Heat map of co-expression gene can be analyzed in the data mining of The Cancer Genome Atlas (TCGA) by UCSC Xena browser.

## Bioinformatics analysis

The bioinformatics data on the miRNA expression and mutation status of PTC were publicly available from UCSC Xena Browser under GDC TCGA Thyroid Cancer (THCA) datasets. Data on miR-222-3p expression was downloaded in file named “TCGA-THCA.mirna.tsv” and data on mutation status was downloaded in file named “TCGA-THCA.mutect2_snv.tsv”. Then, the data of PTC samples in TCGA was extracted and integrated in file named “BrafMutations,csv”. These data was divided into two groups (No mutation and $BRAF^{V600E}$ mutation) according to $BRAF^{V600E}$ mutation status. A two-tailed *t*-test was used for comparing the miRNA expression. $p < 0.05$ was considered as statistically significant. “TCGA-THCA.mirna.tsv”,“TCGA-THCA.mutect2_snv.tsv”, and “BrafMutations,csv” files were uploaded as supplementary data.
